# Objective function estimation for solving optimization problems in gate-model quantum computers

**DOI:** 10.1038/s41598-020-71007-9

**Published:** 2020-08-26

**Authors:** Laszlo Gyongyosi

**Affiliations:** 1 School of Electronics and Computer Science, University of Southampton, Southampton, SO17 1BJ UK; 2 Department of Networked Systems and Services, Budapest University of Technology and Economics, Budapest, 1117 Hungary; 3 MTA-BME Information Systems Research Group, Hungarian Academy of Sciences, Budapest, 1051 Hungary

**Keywords:** Mathematics and computing, Computer science, Pure mathematics

## Abstract

Quantum computers provide a valuable resource to solve computational problems. The maximization of the objective function of a computational problem is a crucial problem in gate-model quantum computers. The objective function estimation is a high-cost procedure that requires several rounds of quantum computations and measurements. Here, we define a method for objective function estimation of arbitrary computational problems in gate-model quantum computers. The proposed solution significantly reduces the costs of the objective function estimation and provides an optimized estimate of the state of the quantum computer for solving optimization problems.

## Introduction

Quantum computers exploit the fundamentals of quantum mechanics to solve computational problems more efficiently than traditional computers^1–20^. Quantum computers can solve computational problems by exploiting the phenomena of quantum superposition and quantum entanglement^5,7–9,18–57^. In a quantum computer, computations are performed on quantum states that carry the information. Gate-model^5,13–18,21,25,43^ quantum computations provide a flexible framework for the realization of quantum computations in the practice. In a gate-model quantum computer, computations are realized by quantum gates (unitary operators); and the quantum-gate architecture integrates a different number of levels and application rounds^5^ to realize gate-model quantum computations^5,18,21,25,36–39,43,58–61^. The output quantum state of the quantum computer is practically measured by a physical measurement apparatus^62–68^ that produces a classical string. In gate-model quantum computers, the quantum states are represented by qubits, the unitaries are realized by qubit gates, and the measurement apparatus is designed for the measurement of qubit systems^13–17,19,69–74^. Another fundamental application scenario of gate-model quantum computations is the small and medium-scale near-term quantum devices of the quantum Internet^69–128^.

An important application scenario of gate-model quantum computers is the maximization of the objective function of computational problems^5,18,21,25,43^. The quantum computer produces a quantum state that yields a high value of the objective function (The objective function subject of a maximization refers to an objective function of an arbitrary computational problem fed into the quantum computer. Objective function examples can be found in^9,24^.). The output state of the quantum computer is measured in a computational basis, and from the measurement result, a classical objective function is evaluated. To get a high-precision estimate of the objective function of the quantum computer, the measurements have to be repeated several times in the physical layer. In each measurement round, a given number of measurement units are applied to measure the output state of the quantum computer. This state represents an objective function value via the quantum-gate attributes in the gate structure of the quantum computer. The objective function values obtained in the measurement rounds are averaged to estimate the objective function of the quantum computer. Since each round requires the preparation of a new quantum state and the application of a high number of measurement units, a high-precision approximation of the objective function value of the quantum computer is a costly procedure. The high-resource assumptions include not just the preparation of the initial and final states of the quantum computer, the application of the unitaries in several rounds, but also the physical apparatus required to measure the output state of the quantum computer. The procedure of the objective function estimation in gate-model quantum computers is therefore a subject of optimization.

Here, we propose a method for the optimized objective function estimation of the quantum computer and for the optimized preparation of the new quantum state of the quantum computer (The terminology “quantum state of the quantum computer” refers to the actual gate parameter values of the unitaries of the quantum computer^5^. Preparation of the target quantum state of the quantum computer refers to the determination of the target gate parameters of the unitaries of the quantum computer.). The framework integrates an objective function extension procedure, a quantum-gate structure segmentation stage, and a machine-learning^11,12,19,50,129–135^ unit called quantum-gate parameter randomization machine learning (QGPR-ML), which outputs the prediction of the new quantum computer state. The aim of the objective function extension is to increase the precision the objective function estimation procedure. An imaginary measurement round refers to a logical measurement round yielded by the post-processing. An imaginary measurement round requires no physical-layer measurement round, since it is resulted by logical-layer procedures and methods in the post-processing stage. The imaginary measurement round also characterizes the performance of the framework. At a particular number of imaginary rounds, the post-processed objective function becomes equal to an objective function yielded from the same number of “real” (e.g., physically implemented) measurement rounds. An initial objective function is calculated from an arbitrary low number of physical measurement rounds, which is then fed into the objective function extension algorithm of the framework. The extended objective function is then fed into a segmentation procedure that decomposes the quantum-gate structure of the quantum computer with respect to the properties of the quantum gates in the quantum circuit. The gate-based segmentation is rooted in the fact that the gate structure unitaries of the quantum computer determine the objective function and therefore the particular output state of the quantum computer. The results are then forwarded into the QGPR-ML block, which achieves a randomization and rule-learning stage. The aim of the randomization is to construct an optimal set for the learning set and test set selections in rule learning. The rule-learning method outputs a set of optimal rules learned from the input. Finally, a prediction stage is applied to the results to determine a new state of the quantum computer for the next iterations.

The novel contributions of our manuscript are as follows:

1. We define a method for objective function estimation for arbitrary computational problems in gate-model quantum computers.
2. The method reduces the costs of quantum state preparations, quantum computational steps and measurements. The proposed algorithms utilize the measurement results and increase the precision of objective function estimation and maximization via computational steps.
3. The results are convenient for solving optimization problems in experimental gate-model quantum computers and for the near-term quantum devices of the quantum Internet.

This paper is organized as follows. In "Related works” section, the related works are discussed. In “System model and problem statement” section, the machine-learning-based objective function optimization framework is proposed. In “Objective function extension and gate structure decomposition” section, the procedures of the framework are discussed. In Section 5, we study the learning model and the quantum computer state prediction method. A performance evaluation is given in “Performance evaluation” section. Finally, “Conclusion” section concludes the results. Supplemental material is included in the Appendix.

## Related works

The related works are summarized as follows.

On the utilized gate-model quantum computer environment, see^5,18^, and^36,38^.

In^5^, the authors studied the problem of objective function estimation of computational problems fed into the quantum computer. The authors focused on a qubit system with a fixed hardware structure in the physical layer. The input quantum system of the quantum circuit is transformed via a sequence of unitaries, and the qubits of the output quantum system are measured by a measurement array. The result of the measurement produces a classical bitstring that is processed further to estimate the objective function of the quantum computer.

Examples of objective functions for quantum computers can be found in^9^.

A quantum circuit design method for gate-model quantum computers has been defined in^36^. In^37^, a method has been defined for the stabilization of the optimal quantum state of the quantum computer.

A method for the evaluation of objective function connectivity in gate-model quantum computers has been proposed in^33^. An unsupervised machine learning method for quantum gate control in gate-model quantum computers has been defined in^34^. In^35^, a framework has been defined for the circuit depth reduction of gate-model quantum computers.

The technique of dense quantum measurement has been defined in^38^. As it has been proven, the method significantly can reduce the number of physical measurement rounds in a gate-model quantum computer environment. In^39^, a training optimization method has been defined for gate-model quantum neural networks.

For some related works on quantum machine learning, see^12,13,43,46,136–143^. For a detailed summary on these references, we suggest also^39^.

Optimization algorithms are also proved to be useful in various applications. In^144^, the authors proposed a neural network ensemble procedure. The aim of the optimization process is to improve the quality of the neural-network based prediction intervals. The prediction intervals are used to quantify uncertainties and disturbances in neural network-based forecasting. The optimization model utilizes the fundaments of simulated annealing and genetic algorithms.

An overview on experimental optimization approaches was proposed in^145^. In this work, the authors provide an overview on recent developments of fault diagnosis and nature-inspired optimal control of industrial process applications. The fields of fault detection and optimal control have proven various successful theoretical results and industrial applications. This work also contains a review on the recent results in machine learning, data mining, and soft computing techniques connected to the particular research fields.

In^146^, the authors studied the problem of training echo state networks (ESN) that are a special form of recurrent neural networks (RNNs). As an important attribute, the ESN structures can be used for a black box modeling of nonlinear dynamical systems. The authors defined a training method that uses a harmony search algorithm, and analyzed the performance of their approach.

In^147^, the authors defined a model-free sliding mode and fuzzy controllers for a particular problem and subject, called reverse osmosis desalination plants. The paper defines an optimization problem in terms of process controlling and fuzzy method. The authors also studied the performance of their solution.

On genetic algorithms for digital quantum simulations, see^148^. In^149^, a method for the learning of an unknown transformation via a genetic approach was defined. In^150^, the authors proposed an overview of existing approaches on quantum computation.

## System model and problem statement

### System model

In the modeled scenario, the goal is the maximization of an objective function *C* via the quantum computer. The aim of the quantum computer run is to produce a quantum state $${\left| \theta \right\rangle } $$ dominated by computational basis states with a high value of an objective function *C*^5,18^ of a computational problem. The quantum computer has $$N_{tot} $$ total number of the quantum gates (unitaries) that formulates a *QG* (quantum gate) structure. Using the $$N_{tot} $$ unitaries $$U_{1} ,\ldots ,U_{N_{tot} } $$, the *QG* structure of the quantum computer produces an output quantum state $${\left| \theta \right\rangle } $$ as^5^

1 $$\begin{aligned} {\left| \theta \right\rangle } =U_{N_{tot} } \left( \theta _{N_{tot} } \right) U_{N_{tot}-1 } \left( \theta _{N_{tot}-1 } \right) \ldots U_{1} \left( \theta _{1} \right) {\left| \psi _{0} \right\rangle } , \end{aligned}$$

where ${\left| \psi _{0} \right\rangle }$ is an initial state and $\theta$ is the gate-parameter vector

2 $$\begin{aligned} \theta ={{\left( {{\theta }_{1}},\ldots ,{{\theta }_{{{N}_{tot}}}} \right) }^{T}}. \end{aligned}$$

The aim is to select the $\theta$ parameter vector such that the expected value of *C* is maximized; thus, the value of quantum objective function

3 $$\begin{aligned} f\left( \theta \right) =\left\langle \theta \left| C \right. | \theta \right\rangle \end{aligned}$$

is high^5^.

A unitary $U_{j} \left( \theta _{j} \right)$ can be written as^5^

4 $$\begin{aligned} U_{j} \left( \theta _{j} \right) =U\left( B_{j} ,\varphi _{j} \right) =\exp \left( -i\varphi _{j} B_{j} \right) , \end{aligned}$$

where $$B_{j} $$ is a set of Pauli operators associated with the *j*th unitary $$U_{j} $$ of the quantum computer, $$j=1,\ldots ,N_{tot} $$, while $$\varphi _{j} $$ is a continuous parameter, $$\varphi _{j} \ge 0$$, referred to as the gate parameter of unitary $$U_{j}$$.

Let $$N_{G} \left( U_{j} \right) $$ refer to the qubit number associated to gate $$U_{j} $$. Then, the $$\varphi _{j} $$ parameter of an $$N_{G} \left( U_{j} \right) $$-qubit unitary $$U_{j}$$ can be classified with respect to $$N_{G} \left( U_{j} \right) $$ as

5 $$\begin{aligned} {\varphi _{j}} =\left\{ \begin{array}{l} {\alpha _{j} ,{\text{if }}N_{G} \left( U_{j} \right) =1} \\ {\beta _{j} ,{\text{if }}N_{G} \left( U_{j} \right) =2} \\ {\vdots } \\ {\Omega _{j} ,{\mathrm{if }}N_{G} \left( U_{j} \right) =N} \end{array}\right. , \end{aligned}$$

where $$N_{G} \left( U_{j} \right) =1$$ identifies an 1-qubit gate $$U_{j} $$ while $$N_{G} \left( U_{j} \right) =N$$ refers to an *N*-qubit gate $$U_{j} $$.

Without loss of generality, at a given $$B_{j} $$, a particular $$U_{j} $$ is approachable via $$\theta _{j} $$, where

6 $$\begin{aligned} \theta _{j} =\varphi _{j} . \end{aligned}$$

Therefore, the ${\left| \theta \right\rangle }$ state of the quantum computer depends on the gate parameters of the unitaries of the quantum computer, and (4) can also be referred as

7 $$\begin{aligned} U_{j} \left( \theta _{j} \right) =U_{j} \left( \varphi _{j} \right) , \end{aligned}$$

where $\varphi _{j}$ is determined as in (5).

Let $$N\left( \varphi _{j} \right) $$ refer to the total number of occurrences of gate parameter value $$\varphi _{j} $$ in the quantum computer (i.e., the number of quantum gates with a particular $$N_{G} $$ qubit number). Then the state $${\left| \theta \right\rangle } $$ of *QG* (see (1)) is evaluated as

8 $$\begin{aligned} \begin{aligned} \left| \theta \right\rangle =&\left| {{\Omega }_{1,\ldots ,N\left( \Omega \right) }},\ldots ,{{\beta }_{1,\ldots ,N\left( \beta \right) }},{{\alpha }_{1,\ldots ,N\left( \varphi \right) }},C \right\rangle \\ =&\left( U\left( {{\alpha }_{N\left( \varphi \right) }} \right) U\left( {{\beta }_{N\left( \beta \right) }} \right) \ldots U\left( {{\Omega }_{N\left( \Omega \right) }} \right) \right) \\ {}&\ldots \left( U\left( {{\alpha }_{1}} \right) U\left( {{\beta }_{1}} \right) \ldots U\left( {{\Omega }_{1}} \right) \right) \left| s \right\rangle , \end{aligned} \end{aligned}$$

where ${\left| s \right\rangle } ={\textstyle \frac{1}{\sqrt{2^{n} } }} \sum _{z}{\left| z \right\rangle }$, where *n* is the length of string *z* resulted from the physical measurement procedure *M*^5^.

Using (4), the function of (3) can be rewritten as

9 $$\begin{aligned} \begin{aligned} f\left( \theta \right) =&\left\langle {{\Omega }_{1,\ldots ,N\left( \Omega \right) }},\ldots ,{{\beta }_{1,\ldots ,N\left( \beta \right) }},{{\alpha }_{1,\ldots ,N\left( \varphi \right) }},C \right| C \\&\left| {{\Omega }_{1,\ldots ,N\left( \Omega \right) }},\ldots ,{{\beta }_{1,\ldots ,N\left( \beta \right) }},{{\alpha }_{1,\ldots ,N\left( \varphi \right) }},C \right\rangle . \end{aligned} \end{aligned}$$

**Figure 1:**
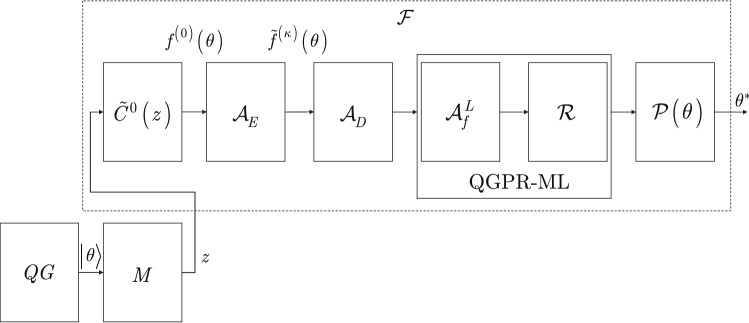
Framework $${{{\mathscr {F}}}}$$ of objective function optimization for gate-model quantum computers. The output $${\left| \theta \right\rangle } $$ of the quantum computer is measured by the *M* measurement that consists of *n* measurement units and yields string *z* and the initial estimate $$f^{\left( 0\right) } \left( \theta \right) $$. At $$R^{*} $$ measurement rounds, the total number of measurements is $$R^{*} n$$. From the measured objective function $${\tilde{C}}^{0} \left( z\right) $$, algorithm $${{{\mathscr {A}}}}_{E} $$ achieves an objective function extension and estimation and outputs $${\tilde{f}}^{\left( \kappa \right) } \left( \theta \right) $$, followed by a feature extraction via algorithm $${{{\mathscr {A}}}}_{D} $$. The QGPR-ML block is decomposed into a randomizing method $${{{\mathscr {A}}}}_{f} $$ applied *L* times (depicted by $${{{\mathscr {A}}}}_{f}^{L} $$) and the $${{{\mathscr {R}}}}$$ rule-generation method. The output of the QGPR-ML block is the $${{{\mathscr {P}}}}\left( \theta \right) $$ prediction of the new value $${{\theta }^{*}}$$ of $$\theta $$.

The schematic model of the objective function optimization framework ${{\mathscr {F}}}$ is depicted in Fig. 1. The notations of the system model are summarized in Table A.1 of the Supplemental Information.

### Problem statement

To get an estimate $f^{\left( 0\right) } \left( \theta \right)$ of function $f\left( \theta \right)$, a measurement *M* is required that yields the *n*-length string *z*, from which $C\left( z\right)$ is calculated. Since *R* measurement rounds required with *n* measurements in each round to get an average objective function ${\tilde{C}}\left( z\right)$

10 $$\begin{aligned} {\tilde{C}}\left( z\right) ={\textstyle \frac{1}{R}} \sum _{i=0}^{R-1}C^{\left( i\right) } \left( z\right) , \end{aligned}$$

where $C^{\left( i\right) } \left( z\right)$, $i=0,\ldots ,R-1$ is an objective function determined in the *i*th round and *z* is the *n*-length string resulted from the measurement of state ${\left| \theta \right\rangle }$ of the quantum computer, it follows that the $\left| M\right|$ total number of required measurements to get the estimate $f^{\left( 0\right) } \left( \theta \right)$ at *R* rounds is

11 $$\begin{aligned} \left| M\right| =Rn. \end{aligned}$$

The problem connected to the objective function estimation is summarized in Problem 1.

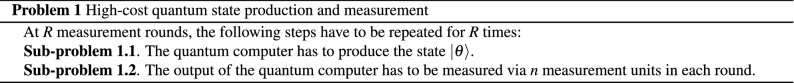



Since each step of Problem 1 is a high-cost procedure, at a given *R*, the cost of the determination of the estimate $f^{\left( 0\right) } \left( \theta \right)$ is significantly high. Here, we show that by setting an arbitrary low number *R* for the number of physical-layer measurement rounds, an arbitrary high-precision estimate $f^{\left( 0\right) } \left( \theta \right)$ can be produced by a well-constructed post-processing stage. Setting $R=1$ represents the situation if only one measurement round is required. The post-processing is referred to as optimization framework ${{{\mathscr {F}}}}$. The results clearly indicate that the number of physical-layer measurements and the number of rounds required by the quantum computer to produce the output quantum state can be significantly decreased by a well-defined post-processing. However, after the *R* measurement rounds are completed, another problem exists, connected to the determination of the new output quantum state $\left| {{\theta }^{*}} \right\rangle$ and summarized in Problem 2.

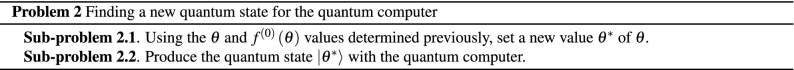



For the solution of Problem 1, we propose algorithm ${{\mathscr {A}}}_{E}$ in the objective function optimization framework ${{{\mathscr {F}}}}$. For the solution of Problem 2, we propose the QGPR-ML procedure in ${{{\mathscr {F}}}}$, which yields the ${{{\mathscr {P}}}}\left( \theta \right)$ prediction for the selection of the new value of $\theta$ for the quantum computer. Since the solution of Problem 1 also eliminates the relevance of Sub-problem 2 of Problem 2, only Sub-problem 1 of Problem 2 remains a challenge.

#### Optimization problems and problem resolutions

The optimization problems connected to the problem resolution are as follows.

1. Define a post-processing framework ${{{\mathscr {F}}}}$ to determine the new optimal state of quantum computer from the measurement results and the parameters of the gate structure of the quantum computer. The problem is resolved via the framework ${{{\mathscr {F}}}}$, ${{{\mathscr {F}}}}:\left\{ {{{\mathscr {A}}}}_{E} ,{{{\mathscr {A}}}}_{D} ,{{{\mathscr {A}}}}_{f}^{L} ,{{\mathscr {R}}},{{{\mathscr {P}}}}\right\}$, that integrates data extension ${{{\mathscr {A}}}}_{E}$, data analytics ${{{\mathscr {A}}}}_{D}$, feature extraction and classification ${{{\mathscr {A}}}}_{f}^{L}$, learning rule generation ${{{\mathscr {R}}}}$ and predictive analytics ${{{\mathscr {P}}}}$.
2. At a given number of $$R^{*} $$ physical measurement rounds, determine the $${\tilde{C}}\left( z\right) $$ objective function that can be estimated after $$\kappa ^{2} R^{*} $$ physical measurement rounds if no post-processing is applied, where $$\kappa \ge 1$$ is a scaling coefficient. The number $$R^{*} $$ of physical measurement rounds cannot be increased, only the measurement results and the available system parameterization of the quantum computer can be used. This optimization problem is resolved via algorithm $${{\mathscr {A}}}_{E} $$ within $${{{\mathscr {F}}}}$$.
3. Determine the $\theta ^{*}$ novel gate-parameter vector via predictive analytics to set the ${\left| \theta ^{*} \right\rangle }$ new state of the quantum computer. This optimization problem is resolved via algorithms ${{{\mathscr {A}}}}_{D} ,{{\mathscr {A}}}_{f}^{L} ,{{{\mathscr {R}}}}$ and ${{{\mathscr {P}}}}$ within ${{{\mathscr {F}}}}$.

### Objective function optimization framework

#### Proposition 1

${{{\mathscr {F}}}}$*is a machine-learning-based objective function optimization framework that determines* $f\left( \theta \right)$*and a new state* ${\left| {{\theta }^{*}} \right\rangle }$*of the quantum computer*.

#### Proof

The input and output and the steps of the proposed machine-learning-based objective function optimization framework ${{{\mathscr {F}}}}$ are described in Procedure 1. The related algorithms and procedures are detailed in the next sections.

The optimization framework therefore yields Output 1 via Step 1 and Output 2 via Step 4 as follows.
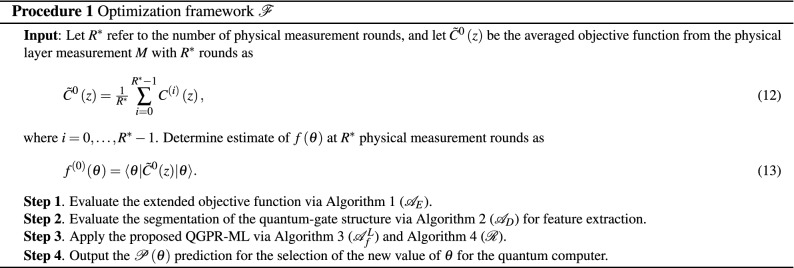


Output 1 is the estimate ${\tilde{f}}^{\left( \kappa \right) } \left( \theta \right)$ of $f\left( \theta \right)$ as

14 $$\begin{aligned} {{{\tilde{f}}}^{( \kappa )}}( \theta )=\langle \theta | {\tilde{C}}( z ) | \theta \rangle , \end{aligned}$$

where ${\tilde{C}}\left( z\right)$ is the averaged objective function

15 $$\begin{aligned} {\tilde{C}}\left( z\right) ={\textstyle \frac{1}{R^{\left( \kappa \right) } }} \sum _{i=0}^{R^{\left( \kappa \right) } -1}C^{\left( i\right) } \left( z\right) , \end{aligned}$$

where $R^{\left( \kappa \right) }$ is the “imaginary” measurement rounds of the post-processing

16 $$\begin{aligned} R^{\left( \kappa \right) } =\kappa ^{2} R^{*} , \end{aligned}$$

where $\kappa$ is a scaling coefficient, defined as

17 $$\begin{aligned} \kappa =\sqrt{\tfrac{{{R}^{\left( \kappa \right) }}}{{{R}^{*}}}}, \end{aligned}$$

while $$R^{*} $$ is the total number of physical measurements, $$R^{\left( \kappa \right) } \ge R^{*} $$, and $$C^{\left( i\right) } \left( z\right) $$ refers to the objective function of the *i*th round, $$i=0,\ldots ,R^{\left( \kappa \right) } -1$$.

Output 2 is the ${{{\mathscr {P}}}}\left( \theta \right)$ prediction for the selection of the new value of $\theta$ to produce new state ${\left| \theta \right\rangle }$ via the quantum computer.

In the ${{{\mathscr {A}}}}_{D}$ segmentation stage, the *QG* quantum circuit of the quantum computer is simplified by preserving the important characteristic of the state of the quantum computer. The segmented values are fed into the QGPR-ML block. The features, like the objective function values, are computed from the segmented gate parameters. The classification of the ${\left| \theta \right\rangle }$ state of the quantum computer is based on the segmented quantum-gate structure. The output of the QGPR-ML block is a new value of $\theta$.

The algorithms (${{{\mathscr {A}}}}_{E}$, ${{{\mathscr {A}}}}_{D}$, ${{{\mathscr {A}}}}_{f}^{L}$, ${{{\mathscr {R}}}}$, ${{{\mathscr {P}}}}$) defined within ${{{\mathscr {F}}}}$ are convergent and operate in an iterative manner such that the outputs converge to specific values. The output of ${{{\mathscr {F}}}}$ at a given initial $\theta$ gate-parameter vector (see (2)) converges to the $\theta ^{*}$ global optimum gate-parameter vector that maximizes the objective function of the quantum computer. $\square$

## Objective function extension and gate structure decomposition

The post-processing framework ${{{\mathscr {F}}}}$ is applied to the results of the *M* measurement procedure that measures the ${\left| \theta \right\rangle }$ state produced by the quantum computer. First, the ${{{\mathscr {A}}}}_{E}$ objective function extension algorithm is applied, followed by the ${{{\mathscr {A}}}}_{D}$ decomposition algorithm. The results are then forwarded to the QGPR-ML machine-learning unit to predict the new state of the quantum computer.

### Objective function extension

#### Theorem 1

*The objective function of the quantum computer can be extended by the* ${{{\mathscr {A}}}}_{E}$*objective function extension algorithm of* ${{{\mathscr {F}}}}$.

#### Proof

Let $$C^{0} \left( z\right) $$ refer to the cumulative objective function resulted from the physical measurement *M* at $$R^{*} $$ rounds and *n* measurements in each rounds as

18 $$\begin{aligned} C^{0} \left( z\right) =\sum _{x=0}^{R^{*} -1}\sum _{y=0}^{n-1}C^{0} \left( x,y\right) , \end{aligned}$$

where $C^{0} \left( x,y\right)$ identifies a component of $C^{\left( 0\right) } \left( z\right)$ obtainable by the measurement of the *y*th qubit, $y=0,\ldots ,n-1$, in the *x*th measurement round, $x=0,\ldots ,R^{*} -1$.

The $d_{C^{0} \left( z\right) }$ dimension (The ${{d}_{X}}$ dimension of *X* refers to the product of the measurement rounds and the measured quantum states per measurement rounds required for the evaluation of *X*.) of $C^{0} \left( z\right)$ is

19 $$\begin{aligned} d_{C^{0} \left( z\right) } =\left( R^{*} \times n\right) . \end{aligned}$$

For the particular $$R^{*} $$ physical measurement rounds, set $$R^{\left( \kappa \right) } $$ as given in (16) with the $$\kappa $$ scaling coefficient.

Since the physical measurement *M* consists of the measurements of *n* qubits, ${\tilde{C}}\left( z\right)$ from (15) can be rewritten as

20 $$\begin{aligned} {\tilde{C}}\left( z\right) =\frac{1}{R^{\left( \kappa \right) } } C^{E} \left( z\right) , \end{aligned}$$

where $C^{E} \left( z\right)$ is the extended objective function defined as

21 $$\begin{aligned} C^{E} \left( z\right) =\sum _{i=0}^{R^{\left( \kappa \right) } -1}C^{\left( i\right) } \left( z\right) =\sum _{x=0}^{R^{\left( \kappa \right) } -1}\sum _{y=0}^{n-1}C\left( x,y\right) , \end{aligned}$$

where $C\left( x,y\right)$ identifies a component of $C^{\left( i\right) } \left( z\right)$ obtainable by the measurement of the *y*th qubit, $y=0,\ldots ,n-1$, in the *x*th measurement round, $x=0,\ldots ,R^{\left( \kappa \right) } -1$, $d_{C^{\left( i\right) } \left( z\right) } =\left( 1 \times n\right)$.

The dimension of $C^{E} \left( z\right)$ is

22 $$\begin{aligned} d_{C^{E} \left( z\right) } =\left( \kappa ^{2} R^{*} \times n\right) . \end{aligned}$$

In our model, the number of “real” physical measurement rounds $$R^{*} $$ is also referred to as the 0th level of “imaginary” measurement $$R^{\left( 0\right) } $$ of the post-processing procedure; thus,

23 $$\begin{aligned} R^{*} =R^{\left( 0\right) } . \end{aligned}$$

Therefore, at a particular $\kappa$, the $R^{\left( \kappa \right) }$ values of *C* are averaged to yield the estimate function ${\tilde{f}}^{\left( \kappa \right) } \left( \theta \right)$ via (14) using ${\tilde{C}}\left( z\right)$ as given in (20), which yields ${\tilde{f}}^{\left( \kappa \right) } \left( \theta \right)$ as

24 $$\begin{aligned} {\tilde{f}}^{(\kappa )} (\theta )={\langle \theta |({\textstyle \frac{1}{R^{(\kappa )} }} C^{E} (z)) \mathrel {| } \theta \rangle } , \end{aligned}$$

where $C^{E} \left( z\right)$ is given in (21).

The discrete wavelet transform is a useful tool in image processing for noise reduction and to enhance the resolution of low-resolution images to obtain high-resolution images^129,130^. Motivated by these features, we show that we can utilize the wavelet transform for the extension of the objective function of the quantum computer. However, in our application framework, both the environment and the aims of the procedure are completely different.

Let ${{{\mathscr {W}}}}\left( C^{\left( i\right) } \left( z\right) \right)$ be the discrete wavelet transform function of the $\left( R^{*} \times n\right)$ dimensional function $C^{\left( i\right) } \left( z\right)$ as

25 $$\begin{aligned} \begin{aligned} \mathscr {W}\left( {{C}^{\left( i \right) }}\left( z \right) \right)&=\frac{1}{\sqrt{{{R}^{*}}n}}\sum \limits _{x=0}^{{{R}^{*}}-1}{\sum \limits _{y=0}^{n-1}{C\left( x,y \right) }}{{f}_{\phi }}\left( x,y \right) \\&=\sum \limits _{j=0}^{{{w}^{\left( l \right) }}-1}{{{W}^{\left( j \right) }}\left( z \right) }, \end{aligned} \end{aligned}$$

where $f_{\phi } \left( \cdot \right)$ are wavelet basis functions, $W^{\left( j\right) } \left( z\right)$ is the transformed objective function, $j=0,\ldots ,w^{\left( l\right) } -1$, where $w^{\left( l\right) }$ is the number of transformed objective function values at a given level *l*, $l\ge 1$, $w^{\left( l\right) } =4+3\left( l-1\right)$, which follows from the execution of ${{{\mathscr {W}}}}$ in (25). The dimension of ${{{\mathscr {W}}}}\left( C^{\left( i\right) } \left( z\right) \right)$ is $d_{{{{\mathscr {W}}}}\left( C^{\left( i\right) } \left( z\right) \right) } =\left( R^{*} \times n\right)$.

Applying the inverse function ${{{\mathscr {W}}}}^{-1} \left( \cdot \right)$ on (25) at a particular $f_{\phi } \left( \cdot \right)$, a given $C^{\left( i\right) } \left( z\right)$ can be expressed as

26 $$\begin{aligned} \begin{aligned} {{C}^{\left( i \right) }}\left( z \right)&={{\mathscr {W}}^{-1}}\left( \mathscr {W}\left( {{C}^{\left( i \right) }}\left( z \right) \right) \right) \\&={{\mathscr {W}}^{-1}}\left( \sum \limits _{j=0}^{{{w}^{\left( l \right) }}-1}{{{W}^{\left( j \right) }}\left( z \right) } \right) \\&=\frac{1}{\sqrt{{{R}^{*}}n}}\sum \limits _{x=0}^{{{R}^{*}}-1}{\sum \limits _{y=0}^{n-1}{\mathscr {W}\left( {{C}^{\left( i \right) }}\left( z \right) \right) }}{{f}_{\phi }}\left( x,y \right) . \end{aligned} \end{aligned}$$

The proposed method for the objective function extension is given in Algorithm 1 ($${{{\mathscr {A}}}}_{E} $$). Algorithm 1 integrates Sub-procedure 1 ($$P_{E} $$) for the objective function extension.

The description of Sub-Procedure 1 ($$P_{E} $$) is as follows.

These results conclude the proof. $\square$

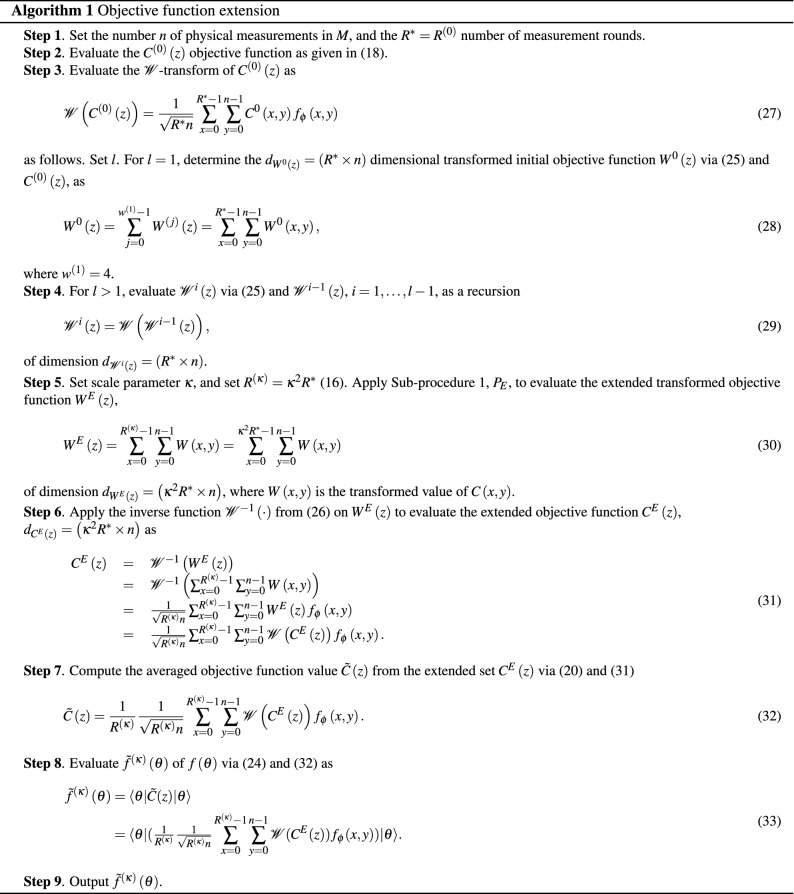

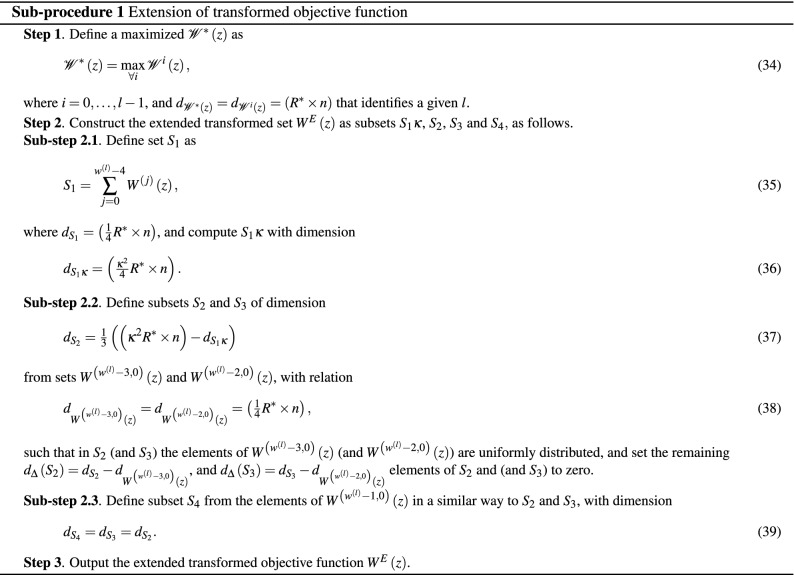





#### Lemma 1

*The precision of the estimation of the objective function yielded from a physical-layer measurement* *M* *can be improved via the* ${{{\mathscr {A}}}}_{E}$*objective function extension algorithm of* ${{{\mathscr {F}}}}$.

#### Proof

In algorithm $${{{\mathscr {A}}}}_{E} $$, function $${{\mathscr {W}}}^{-1} \left( \cdot \right) $$ applied on $$W^{E} \left( z\right) $$ yields the extended objective function $$C^{E} \left( z\right) $$, from which estimate $${\tilde{f}}^{\left( \kappa \right) } \left( \theta \right) $$ of $$f\left( \theta \right) $$ can be determined at $$R^{*} $$ physical measurement rounds. The produced estimate $${\tilde{f}}^{\left( \kappa \right) } \left( \theta \right) $$ is equivalent to the estimate $$f^{\left( 0\right) } \left( \theta \right) $$ obtainable at $$R^{\left( \kappa \right) } =\kappa ^{2} R^{*} $$ physical measurement rounds, with $$\left| M\right| =n\kappa ^{2} R^{*} $$ total measurements. The details are as follows. Since the dimension of $$W^{E} \left( z\right) $$ is $$d_{W^{E} \left( z\right) } =\left( \kappa ^{2} R^{*} \times n\right) $$, the $$C^{E} \left( z\right) $$ extended objective function values contains $$R^{\left( \kappa \right) } =\kappa ^{2} R^{*} $$ (16) objective functions evaluated for each measurement round. The estimate $${\tilde{f}}\left( \theta \right) $$ yielded by the application of $${{{\mathscr {W}}}}^{-1} \left( \cdot \right) $$ on $$W^{E} \left( z\right) $$ is analogous to the estimate $$f^{\left( 0\right) } \left( \theta \right) $$ that can be extracted by $$\left| M\right| $$ number of measurements in the physical-layer measurement apparatus *M* via $$R^{\left( \kappa \right) } $$ measurement rounds as

40 $$\begin{aligned} \left| M\right| =\kappa ^{2} \left| M^{*} \right| =\kappa ^{2} nR^{*} , \end{aligned}$$

where $\left| M^{*} \right| =nR^{*}$ is the total number of physical-layer measurements. The proof is concluded here. $\square$

#### Objective function extension factor

Let $$C^{0} \left( z\right) $$ be the objective function resulting from the $$R^{*} $$ measurement rounds with dimension $$d_{C^{0} \left( z\right) } =\left( R^{*} \times n\right) $$, where $$C^{0} \left( z\right) $$ is given in (18), $${{\mathscr {W}}}\left( C^{0} \left( z\right) \right) $$ and $$W^{E} \left( z\right) ={{{\mathscr {W}}}}^{-1} \left( {{{\mathscr {W}}}}\left( C^{0} \left( z\right) \right) \right) $$ be the transformed and extended transformed objective function with dimensions $$d_{W^{0} \left( z\right) } =\left( R^{*} \times n\right) $$ and $$d_{W^{E} \left( z\right) } =\left( \kappa ^{2} R^{*} \times n\right) $$ as given in (28) and (30), and $$C^{E} \left( z\right) $$ be the extended objective function (see (31)) with dimension $$d_{C^{E} \left( z\right) } =\left( \kappa ^{2} R^{*} \times n\right) $$.

Then let $\lambda _{E} \left( \cdot \right)$ be the objective function extension factor, defined as

41 $$\begin{aligned} \begin{aligned} \lambda _{E} \left( W^{E} \left( z\right) ,C^{E} \left( z\right) \right)&={\textstyle \frac{\sum _{x=0}^{R^{\left( \kappa \right) } -1}\sum _{y=0}^{n-1}\left( C\left( x,y\right) -W\left( x,y\right) \right) ^{2} }{\sum _{x=0}^{R^{*} -1}\sum _{y=0}^{n-1}\left( C^{0} \left( x,y\right) -W^{0} \left( x,y\right) \right) ^{2} }}. \end{aligned} \end{aligned}$$

The quantity in (41) therefore identifies the ratio of the difference of the extended objective function and the extended transformed objective function and the difference of the initial objective function and the initial extended objective function.

### Quantum-gate structure decomposition

#### Theorem 2

The ${\left| \theta \right\rangle }$ state of the quantum computer is decomposable by the $\varphi$ gate parameters of the quantum computer.

#### Proof

The proposed scheme can be applied for an arbitrary *d*-dimensional quantum-gate structure; however, for simplicity, we assume the use of qubit gates. Thus, in the *QG* structure of the quantum computer, we set $d=2$ for the dimension of the quantum gates. Since the $\varphi$ gate parameters determine the state ${\left| \theta \right\rangle }$ of the quantum computer (8), the segmentation of the quantum-gate structure is based on the $\varphi$ gate parameters.

Let $$N_{G} \left( U_{j} \right) $$ refer to the qubit number associated with gate $$U_{j} $$, and let $$\varphi _{j} $$ be a gate parameter of an $$N_{G} \left( U_{j} \right) $$-qubit gate unitary $$U_{j} \left( \varphi _{j} \right) $$ as given in (5).

Let $$n_{t} $$ be the number of classes selected for the segmentation of the $$\varphi $$ gate parameters of the *QG* structure of the quantum computer. Let $$H_{k} $$ be the entropy function associated with the *k*th class, $$k=1,\ldots ,n_{t} $$, and $$f(\vec {\phi })$$ be the objective function of the segmentation of the *QG* structure as

42 $$\begin{aligned} f(\vec {\phi })=\sum _{k=1}^{n_{t} }H_{k} , \end{aligned}$$

where $\vec {\phi }$ is an $d_{\vec {\phi }} =\left( n_{t} -1\right)$-dimensional vector $\vec {\phi }=\left[ \phi _{1} ,\ldots ,\phi _{n_{t} -1} \right]$, where $\phi _{l}$ is the gate segmentation parameter to classify the $\varphi$ gate parameters into *l*th and $\left( l+1\right)$-th classes, such that

43 $$\begin{aligned} 0\le \phi _{l} \le \chi , \end{aligned}$$

where $\chi$ is an upper bound on the $\varphi _{i}$ gate parameters of the quantum computer,

44 $$\begin{aligned} \mathop {\max }\limits _{\forall i} \varphi _{i} \le \chi . \end{aligned}$$

Let $\vec {\phi }^{*}$ be the optimal vector that maximizes the overall entropy in (42),

45 $$\begin{aligned} \vec {\phi }^{*} =\left[ \phi _{1}^{*} ,\ldots ,\phi _{n_{t} -1}^{*} \right] , \end{aligned}$$

with $\left( n_{t} -1\right)$ optimal parameters, $0\le \phi _{l}^{*} \le \chi$; $l=1,\ldots ,n_{t} -1$ subject to be determined as

46 $$\begin{aligned} \vec {\phi }^{*} =\arg \mathop {\max }\limits _{\vec {\phi }} f(\vec {\phi }), \end{aligned}$$

which yields the maximization of the $f\left( \vec {\phi }^{*} \right)$ objective function (42).

The $$H_{k} $$ entropies in (42) are defined as

47 $$\begin{aligned} H_{k} =\left\{ \begin{array}{l} {H_{1} =\sum _{i=1}^{\phi _{1}^{*} }{\textstyle \frac{\Pr \left( N\left( \varphi _{i} \right) \right) }{\omega _{1} }} \ln \left( {\textstyle \frac{\Pr \left( N\left( \varphi _{i} \right) \right) }{\omega _{1} }} \right) {\text{, if }}k=1 } \\ {H_{2} =\sum _{i=\phi _{1}^{*} +1}^{\phi _{2}^{*} }{\textstyle \frac{\Pr \left( N\left( \varphi _{i} \right) \right) }{\omega _{2} }} \ln \left( {\textstyle \frac{\Pr \left( N\left( \varphi _{i} \right) \right) }{\omega _{2} }} \right) {\text{, if }}k=2 } \\ {\vdots } \\ {H_{n_{t} } =\sum _{i=\phi _{n_{t} -1}^{*} +1}^{\chi }{\textstyle \frac{\Pr \left( N\left( \varphi _{i} \right) \right) }{\omega _{n_{t} } }} \ln \left( {\textstyle \frac{\Pr \left( N\left( \varphi _{i} \right) \right) }{\omega _{n_{t} } }} \right) ,{\text{if }}k=n_{t} } \end{array}\right. , \end{aligned}$$

where $N\left( \varphi _{i} \right)$ is the number of occurrences of gate parameter $\varphi _{i}$ in the *QG* structure, with probability distribution $\Pr \left( N\left( \varphi _{i} \right) \right)$ as

48 $$\begin{aligned} \Pr \left( N\left( \varphi _{i} \right) \right) ={\textstyle \frac{N\left( \varphi _{i} \right) }{N_{tot} }} , \end{aligned}$$

where $$N_{tot} $$ is the total number of quantum gates in the quantum computer,

49 $$\begin{aligned} \sum _{i=1}^{N_{tot} }\Pr \left( N\left( \varphi _{i} \right) \right) =1, \end{aligned}$$

while $\omega _{i}$s are sum-of-probability distributions, as

50 $$\begin{aligned} \omega _{QG} =\left\{ \begin{array}{l} {\omega _{1} =\sum _{i=1}^{\phi _{1}^{*} }\Pr \left( N\left( \varphi _{i} \right) \right) } \\ {\omega _{2} =\sum _{i=\phi _{1}^{*} +1}^{\phi _{2}^{*} }\Pr \left( N\left( \varphi _{i} \right) \right) } \\ {\vdots } \\ {\omega _{n_{t} } =\sum _{i=\phi _{n_{t} -1}^{*} +1}^{\chi }\Pr \left( N\left( \varphi _{i} \right) \right) } \end{array}\right. . \end{aligned}$$

Using (48) and (50), the *QG* structure can be segmented into $$n_{t} $$ classes, $$\mathscr {C}{{}_{QG}}:\left\{ \mathscr {C}{{}_{1}},\ldots ,\mathscr {C}{{}_{{{n}_{t}}}} \right\} $$ as

51 $$\begin{aligned} \mathscr {C}{{}_{QG}} =\left\{ \begin{array}{l} {\mathscr {C}{{}_{1}} ={\textstyle \frac{\Pr \left( N\left( \varphi _{1} \right) \right) }{\omega _{1} }} ,\ldots ,{\textstyle \frac{\Pr \left( N\left( \varphi _{\phi _{1}^{*} } \right) \right) }{\omega _{1} }} } \\ {\mathscr {C}{{}_{2}} ={\textstyle \frac{\Pr \left( N\left( \varphi _{\phi _{1}^{*} +1} \right) \right) }{\omega _{2} }} ,\ldots ,{\textstyle \frac{\Pr \left( N\left( \varphi _{\phi _{2}^{*} } \right) \right) }{\omega _{2} }} } \\ {\vdots } \\ {\mathscr {C}{{}_{{{n}_{t}}}} ={\textstyle \frac{\Pr \left( N\left( \varphi _{\phi _{n_{t} -1}^{*} +1} \right) \right) }{\omega _{n_{t} } }} ,\ldots ,{\textstyle \frac{\Pr \left( N\left( \varphi _{\chi } \right) \right) }{\omega _{n_{t} } }} } \end{array}\right. , \end{aligned}$$

with class mean values $\mu {{}_{QG}}:\left\{ \mu {{}_{1}},\ldots ,{{\mu }_{{{n}_{t}}}} \right\}$ as

52 $$\begin{aligned} \mu {{}_{QG}} =\left\{ \begin{array}{l} {\mu _{1} =\sum _{i=1}^{\phi _{1}^{*} }{\textstyle \frac{i\Pr \left( N\left( \varphi _{i} \right) \right) }{\omega _{1} }} } \\ {\mu _{2} =\sum _{i=\phi _{1}^{*} +1}^{\phi _{2}^{*} }{\textstyle \frac{i\Pr \left( N\left( \varphi _{i} \right) \right) }{\omega _{2} }} } \\ {\vdots } \\ {\mu _{n_{t} } =\sum _{i=\phi _{n_{t} -1}^{*} +1}^{\chi }{\textstyle \frac{i\Pr \left( N\left( \varphi _{i} \right) \right) }{\omega _{n_{t} } }} } \end{array}\right. . \end{aligned}$$

As the objective function and the related quantities are determined by Algorithm 2 (${{{\mathscr {A}}}}_{D}$), a particular gate parameter $\varphi _{j}$ is therefore classified as

53 $$\begin{aligned} \mathscr {C}{{}_{QG}} \in \varphi _{j} =\left\{ \begin{array}{l} {\mathscr {C}{{}_{1}} \in \varphi _{j} , {\text{ if 0}}\le \varphi _{j}<\phi _{1}^{*} ,} \\ {\mathscr {C}{{}_{2}} \in \varphi _{j}, {\text{ if }}\phi _{1}^{*} \le \varphi _{j}<\phi _{2}^{*} ,} \\ {\vdots } \\ {\mathscr {C}{{}_{{{n}_{t}}}} \in \varphi _{j} ,{\text{ if }}\phi _{n_{t} -1}^{*} \le \varphi _{j} <\chi .} \end{array}\right. \end{aligned}$$

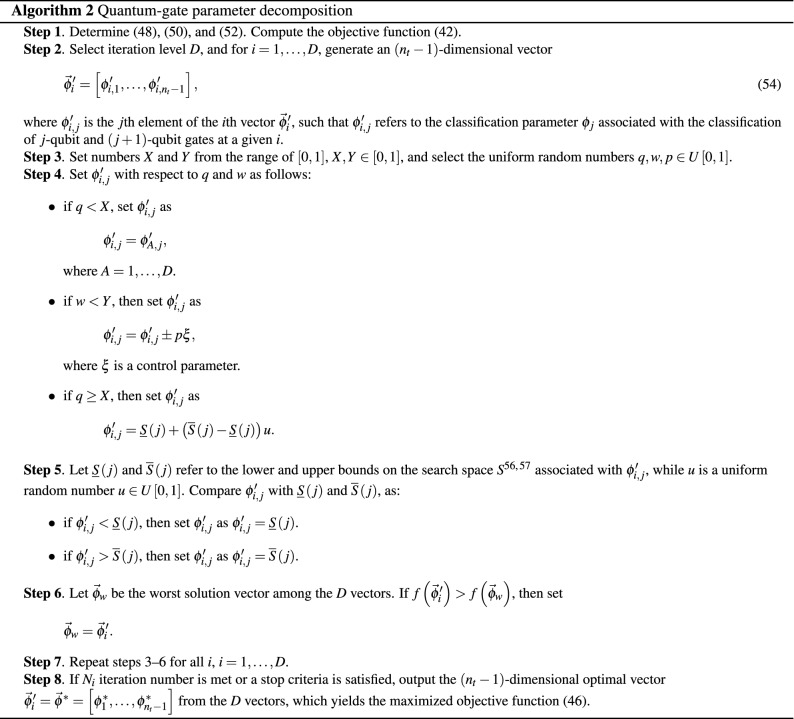
Motivated by the multilevel segmentation procedures^131,132^, the steps of ${{{\mathscr {A}}}}_{D}$ are given in Algorithm 2.

According to Algorithm 2, the $\phi '_{i,j}$ gate classification parameter is evaluated via events ${{E}_{i}}$ as

55 $$\begin{aligned} \phi '_{i,j} =\left\{ \begin{array}{l} {{{E}_{1}}:\phi '_{A,j} ,A=1,\ldots ,D} \\ {{{E}_{2}}:{\underline{S}} \left( j\right) +\left( {\overline{S}}\left( j\right) -{\underline{S}} \left( j\right) \right) u} \\ {{{E}_{3}}:\phi '_{i,j} \pm p\xi } \\ {{{E}_{4}}:\phi '_{i,j} } \end{array}\right. , \end{aligned}$$

with the related probabilities^132^

56 $$\begin{aligned} \Pr \left( \phi '_{i,j} \right) =\left\{ \begin{array}{l} {\Pr \left( E_{1} \right) =X} \\ {\Pr \left( E_{2} \right) =1-X} \\ {\Pr \left( E_{3} \right) =Y} \\ {\Pr \left( E_{4} \right) =1-Y} \end{array}\right. . \end{aligned}$$

The proof is concluded here. $\square$

#### Error of gate-parameter decomposition

The $\varepsilon _{\vec {\phi }^{*} }$ error associated with the gate-parameter segmentation algorithm ${{{\mathscr {A}}}}_{D}$ at a given $\vec {\phi }^{*}$, $\varepsilon _{\vec {\phi }^{*} }$ is defined as

57 $$\begin{aligned} \varepsilon _{\vec {\phi }^{*} } =\sqrt{{\textstyle \frac{\sum _{i=0}^{D_{QG} -1}\sum _{j=0}^{n-1}\left( \varphi _{QG_{R} } \left( i,j\right) -\varphi _{QG}^{\vec {\phi }^{*} } \left( i,j\right) \right) }{D_{QG} n}} } , \end{aligned}$$

where $$D_{QG} $$ is the depth of the quantum circuit *QG* of the quantum computer, *n* is the number of measurement blocks at the *QG* circuit output, $$\varphi _{QG_{R} } \left( i,j\right) $$ is the $$\varphi $$ gate parameter associated with the $$\left( i,j\right) $$-th gate of a reference quantum circuit $$QG_{R} $$, $$i=0,\ldots ,D_{QG} -1$$, $$j=0,\ldots ,n-1$$, ($$\varphi _{QG}^{R} \left( i,j\right) =0$$ if there is no gate at $$\left( i,j\right) $$ in *QG*), and $$\varphi _{QG}^{\vec {\phi }^{*} } \left( i,j\right) $$ is the $$\varphi $$ gate parameter associated with the $$\left( i,j\right) $$-th gate of the segmented *QG* circuit ($$\varphi _{QG}^{\vec {\phi }^{*} } \left( i,j\right) =0$$ if there is no gate at $$\left( i,j\right) $$ in *QG*).

## Gate parameter randomization machine learning

The QGPL-ML block splits further the results of ${{\mathscr {A}}}_{D}$ to achieve a randomized data partitioning and to generate rules. The QGPL-ML method integrates algorithms ${{\mathscr {A}}}_{f}^{L}$ and ${{{\mathscr {R}}}}$. Algorithm ${{\mathscr {A}}}_{f}^{L}$ is defined for the data randomization and selection for the learning, while algorithm ${{{\mathscr {R}}}}$ is defined for the rule learning.

Motivated by granulated computing^133,134^, the data randomization of ${{{\mathscr {A}}}}_{f}^{L}$ in the QGPL-ML block is based on the gate parameters of the quantum gates. The algorithm selects the best training and test instances for the rule-learning block via a ratio parameter $r\in \left[ 0,1\right]$ in a multilevel structure. As a corollary, ${{{\mathscr {A}}}}_{f}^{L}$ avoids class imbalance and sample representativeness issues^133,134^. Using the results of ${{{\mathscr {A}}}}_{f}^{L}$, the rule-generation procedure ${{{\mathscr {R}}}}$ uses rule-quality metrics (leverage^133–135^) to identify the best rules in each iteration step. The result of ${{\mathscr {R}}}$ is *L* optimal rules, where *L* is the application number (level) of ${{{\mathscr {A}}}}_{f}$.

### Randomization and probability distribution

The benefits of the proposed randomization in ${{\mathscr {A}}}_{f}^{L}$ are as follows. The randomization applied ${{{\mathscr {A}}}}_{f}^{L}$ in allows us to create an optimal ${{{\mathscr {S}}}}_{l}$ learning set and optimal ${{{\mathscr {S}}}}_{t}$ test set in the ${{{\mathscr {R}}}}$ rule learning stage. The optimality means that the input data is partitioned into a learning set and test set in a semi-randomized (granulated^133,134,151,152^) way (i.e., not fully randomized) to avoid the issues of class imbalance and sample representativeness. These problems are connected to a fully randomization^151,152^.

The problem of class imbalance means that the ratio of classes of the constructed learning set and test set do not represent the ratio of classes of the input data. This problem could occur at a non-optimal random partitioning of the input data, and could bring up in both the training and the test set, respectively^133,134,151,152^.

The problem of sample representativeness is an integrity problem, and it refers to the problem if the training and test instances have no any connection, which could lead to inconsistency in the learning process^151,152^.

The procedure of ${{{\mathscr {A}}}}_{f}^{L}$ applies a semi-randomization on the input data, to avoid these issues. The effect of probability distribution of the randomization in ${{{\mathscr {A}}}}_{f}^{L}$ determines the precision of the construction of the training and test sets. The ${{{\mathscr {A}}}}_{f}^{L}$ procedure allows us to keep the class consistency of the input data in the training and test sets, and also to keep the integrity of the instances of the training and test sets. To measure the precision of ${{{\mathscr {A}}}}_{f}^{L}$, we utilized the ${{{\mathscr {L}}}}$ leverage metric^135^, ${{{\mathscr {L}}}}\in \left[ 0,1\right]$ in the ${{{\mathscr {R}}}}$ rule learning stage. The probability distribution in ${{{\mathscr {A}}}}_{f}^{L}$ has effect on the rule precision generated by ${{{\mathscr {R}}}}$ since it uses the outputs of ${{{\mathscr {A}}}}_{f}^{L}$. At a full randomization in ${{{\mathscr {A}}}}_{f}^{L}$, the ${{\mathscr {L}}}$ value in ${{{\mathscr {R}}}}$ low, ${{{\mathscr {L}}}}\rightarrow 0$, while for a semi-randomization in ${{{\mathscr {A}}}}_{f}^{L}$, ${{{\mathscr {L}}}}$ picks up high values, ${{{\mathscr {L}}}}\rightarrow 1$, in ${{{\mathscr {R}}}}$.

### Procedures

The procedure ${{{\mathscr {A}}}}_{f}^{L}$ of the QGPL-ML block is detailed in Algorithm 3.

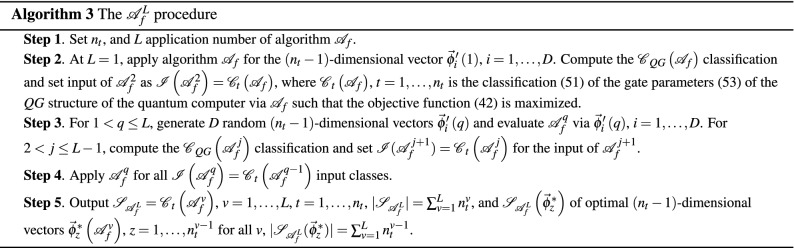



The procedure ${{{\mathscr {R}}}}$ of the QGPL-ML block is detailed in Algorithm 4.

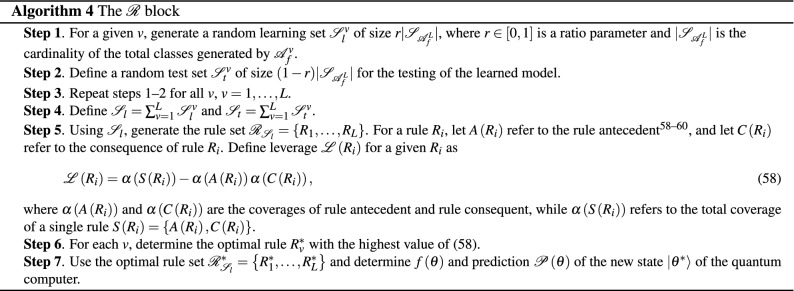



### State of the quantum computer

#### Theorem 3

*The state* ${\left| {{\theta }^{*}} \right\rangle }$*of the quantum computer can be made by the output* ${{{\mathscr {P}}}}\left( \theta \right)$*of the QGPL-ML procedure*.

#### Proof

The $\theta ^{*}$ new gate parameter vector is determined via a ${{{\mathscr {P}}}}$ predictive analytics. The ${{{\mathscr {P}}}}$ unit utilizes the outputs generated by the units ${{\mathscr {A}}}_{E}$, ${{{\mathscr {A}}}}_{D}$, ${{{\mathscr {A}}}}_{f}^{L}$ and ${{{\mathscr {R}}}}$ of ${{{\mathscr {F}}}}$. The input of ${{{\mathscr {A}}}}_{f}^{L}$ is provided by ${{{\mathscr {A}}}}_{D}$ (Algorithm 2), such that the input of ${{{\mathscr {A}}}}_{D}$ is the extended set of gate parameters determined by the extension algorithm ${{{\mathscr {A}}}}_{E}$ (Algorithm 1). The prediction of the $\theta ^{*}$ can be made at an initial $\theta$ as

59 $$\begin{aligned} \theta ^{*} =\theta +\rho , \end{aligned}$$

where $\rho$ is the gate parameter modification vector

60 $$\rho = {\left( {{\alpha _1}, \ldots ,{\alpha _{{N_{tot}}}}} \right)^T},$$

where $\alpha _{i}$ calibrates the gate parameter $\theta _{i}$ of the *i*th unitary, $i=1,\ldots ,N_{tot}$. The actual value of $\alpha _{i}$ depends on the error $\varepsilon _{\vec {\phi }^{*} }$ (57) associated with ${{{\mathscr {A}}}}_{D}$.

The precision of the prediction is also controlled by a $$\tau $$ parameter, which quantifies the minimum of number of classes ($$n_{t} $$) selected for the classification of the quantum-gate parameters in the $${{{\mathscr {A}}}}_{f}^{L} $$ procedure.

As the new gate parameter vector

61 $$\begin{aligned} \theta ^{*} =\left( \left( \theta _{1} +\alpha _{1} \right) ,\ldots ,\left( \theta _{N_{tot} } +\alpha _{N_{tot} } \right) \right) ^{T} \end{aligned}$$

is determined, the quantum computer can set up the state ${\left| \theta ^{*} \right\rangle }$.

The prediction of the ${\left| {{\theta }^{*}} \right\rangle }$ new state of the quantum computer are given in Procedure 2.
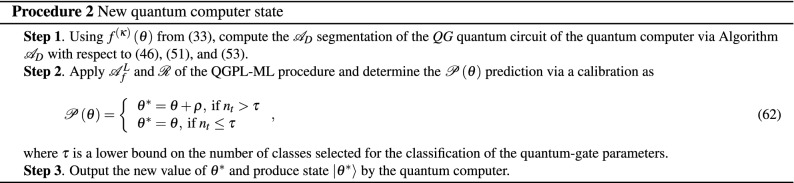


These results conclude the proof. $\square$

## Performance evaluation

This section proposes a performance evaluation for the method validation and comparison. We study the precision of the objective function estimation, the estimation error, and the cost reduction in the objective function estimation process.

### Objective function estimation

et ${\tilde{C}}^{{{{\mathscr {R}}}}} \left( z\right)$ be the ${{{\mathscr {R}}}}$ reference objective function that can be estimated at $R_{{{{\mathscr {R}}}}}^{*}$ reference physical measurement rounds,

63 $$\begin{aligned} R_{{{{\mathscr {R}}}}}^{*} =R^{\left( \kappa \right) } , \end{aligned}$$

as

64 $$\begin{aligned} {\tilde{C}}^{{{{\mathscr {R}}}}} \left( z\right) ={\textstyle \frac{1}{R_{{{{\mathscr {R}}}}}^{*} }} \sum _{i=0}^{R_{{{{\mathscr {R}}}}}^{*} -1}C^{r,\left( i\right) } \left( z\right) ={\textstyle \frac{1}{R_{{{{\mathscr {R}}}}}^{*} }} C^{r} \left( z\right) , \end{aligned}$$

where $$C^{r,\left( i\right) } $$ is the reference objective function evaluated in the *i*th physical measurement round, $$i=0,\ldots ,R_{{{{\mathscr {R}}}}}^{*} -1,$$$$d_{C^{r,\left( i\right) } } =1\times n$$, and $$C^{r} \left( z\right) $$ is the sum of the $$\kappa ^{2} R^{*} $$ reference objective functions, with dimension $$d_{C^{r} \left( z\right) } =d_{C_{E} \left( z\right) } $$, where $$d_{C^{E} \left( z\right) } $$ is as given in (22).

The $R_{{{{\mathscr {R}}}}}^{*}$ number of measurement round serves also as reference to a comparison in the performance evaluation with the scheme of^5^, that utilizes only physical layer measurement (i.e., refers to the case if no post-processing is applied).

Let ${\tilde{C}}\left( z\right)$ be the observed output objective function (see (20)) estimated via the $C_{E} \left( z\right)$ extended objective function (see (21)) at $R^{\left( \kappa \right) }$, as ${\tilde{C}}\left( z\right) ={\textstyle \frac{1}{R^{\left( \kappa \right) } }} \sum _{i=0}^{R^{\left( \kappa \right) } -1}C^{\left( i\right) } \left( z\right) ={\textstyle \frac{1}{R^{\left( \kappa \right) } }} C^{E} \left( z\right)$.

Then, let $\sigma _{{\tilde{C}}^{{{{\mathscr {R}}}}} \left( z\right) }$ be the standard deviation of ${\tilde{C}}^{{{{\mathscr {R}}}}} \left( z\right)$, defined as

65 $$\begin{aligned} \sigma _{{\tilde{C}}^{{{{\mathscr {R}}}}} \left( z\right) } =\left( {\textstyle \frac{1}{R_{{{{\mathscr {R}}}}}^{*} -1}} \sum _{i=0}^{R_{{{{\mathscr {R}}}}}^{*} -1}\left( C^{r,\left( i\right) } -{\tilde{C}}^{{{{\mathscr {R}}}}} \left( z\right) \right) ^{2} \right) ^{{1 /2} } , \end{aligned}$$

and let $\sigma _{{\tilde{C}}\left( z\right) }$ be the standard deviation of ${\tilde{C}}\left( z\right)$, defined as

66 $$\begin{aligned} \sigma _{{\tilde{C}}\left( z\right) } =\left( {\textstyle \frac{1}{R^{\left( \kappa \right) } -1}} \sum _{i=0}^{R^{\left( \kappa \right) } -1}\left( C^{\left( i\right) } \left( z\right) -{\tilde{C}}\left( z\right) \right) ^{2} \right) ^{{1 /2}} , \end{aligned}$$

while ${{\sigma }_{{{{{\tilde{C}}}}^{\mathscr {R}}}\left( z \right) }}_{{\tilde{C}}\left( z \right) }$ is defined^153^ as

67 $$\begin{aligned} {{\sigma }_{{{{{\tilde{C}}}}^{\mathscr {R}}}\left( z \right) }}_{{\tilde{C}}\left( z \right) } ={\textstyle \frac{1}{R_{{{{\mathscr {R}}}}}^{*} -1}} \sum _{i=0}^{R_{{{{\mathscr {R}}}}}^{*} -1}\left( C^{r,\left( i\right) } -{\tilde{C}}^{{{{\mathscr {R}}}}} \left( z\right) \right) \left( C^{\left( i\right) } \left( z\right) -{\tilde{C}}\left( z\right) \right) . \end{aligned}$$

Using (65), (66) and (67), we define the quantity $\Phi ({\tilde{C}}^{{{{\mathscr {R}}}}} \left( z\right) ,{\tilde{C}}\left( z\right) )$ to measure the precision of estimation ${\tilde{C}}\left( z\right)$ at a particular reference objective function ${\tilde{C}}^{{{\mathscr {R}}}} \left( z\right)$, as

68 $$\begin{aligned} \Phi ({\tilde{C}}^{{{{\mathscr {R}}}}} \left( z\right) ,{\tilde{C}}\left( z\right) )={\textstyle \frac{\left( 2{\tilde{C}}^{{{{\mathscr {R}}}}} \left( z\right) {\tilde{C}}\left( z\right) \right) \left( 2{{\sigma }_{{{{{\tilde{C}}}}^{\mathscr {R}}}\left( z \right) }}_{{\tilde{C}}\left( z \right) } \right) }{\left( \left( {\tilde{C}}^{{{{\mathscr {R}}}}} \left( z\right) \right) ^{2} +\left( {\tilde{C}}\left( z\right) \right) ^{2} \right) \left( \sigma _{{\tilde{C}}^{{{{\mathscr {R}}}}} \left( z\right) }^{2} +\sigma _{{\tilde{C}}\left( z\right) }^{2} \right) }} , \end{aligned}$$

where $\Phi ({\tilde{C}}^{{{{\mathscr {R}}}}} \left( z\right) ,{\tilde{C}}\left( z\right) )\in \left[ 0,1\right]$ such that at $\Phi ({\tilde{C}}^{{{{\mathscr {R}}}}} \left( z\right) ,{\tilde{C}}\left( z\right) )=0$, ${\tilde{C}}\left( z\right)$ is completely uncorrelated from the reference objective function ${\tilde{C}}^{{{{\mathscr {R}}}}} \left( z\right)$, while at $\Phi ({\tilde{C}}^{{{{\mathscr {R}}}}} \left( z\right) ,{\tilde{C}}\left( z\right) )=1$ the observed ${\tilde{C}}\left( z\right)$ coincidences with ${\tilde{C}}^{{{{\mathscr {R}}}}} \left( z\right)$.

Note, that from $\Phi ({\tilde{C}}^{{{{\mathscr {R}}}}} \left( z\right) ,{\tilde{C}}\left( z\right) )$ (see (68)) and ${\tilde{C}}\left( z\right)$ (see (20)), the value of ${\tilde{C}}^{{{{\mathscr {R}}}}} \left( z\right)$ can be evaluated as follows. Let

69 $$\begin{aligned} v({\tilde{C}}^{{{{\mathscr {R}}}}} \left( z\right) )=\left( C^{r,\left( 0\right) } ,\ldots ,C^{r,\left( R_{{{{\mathscr {R}}}}}^{*} -1\right) } \right) ^{T} \end{aligned}$$

be a vector formulated from the elements of ${\tilde{C}}^{{{{\mathscr {R}}}}} \left( z\right)$, and let

70 $$\begin{aligned} v({\tilde{C}}\left( z\right) )=\left( C^{\left( 0\right) } \left( z\right) ,\ldots ,C^{\left( R^{\left( \kappa \right) } -1\right) } \left( z\right) \right) ^{T} \end{aligned}$$

be a vector formulated form the elements of ${\tilde{C}}\left( z\right)$.

Then, at a particular $\Phi ({\tilde{C}}^{{{{\mathscr {R}}}}} \left( z\right) ,{\tilde{C}}\left( z\right) )$, the reference $v({\tilde{C}}^{{{{\mathscr {R}}}}} \left( z\right) )$ can be evaluated from $v({\tilde{C}}\left( z\right) )$ in a convergent and iterative manner, as

71 $$\begin{aligned} \begin{aligned} v({\tilde{C}}\left( z\right) )=\,&v({\tilde{C}}\left( z\right) )\pm \chi {{{\mathscr {P}}}}(v({\tilde{C}}^{{{{\mathscr {R}}}}} \left( z\right) ),v({\tilde{C}}\left( z\right) ))\\ {}&\cdot \nabla _{{\tilde{C}}\left( z\right) } (\Phi ({\tilde{C}}^{{{{\mathscr {R}}}}} \left( z\right) ,{\tilde{C}}\left( z\right) )), \end{aligned} \end{aligned}$$

where $\chi$ is a coefficient^153^, $\nabla _{{\tilde{C}}\left( z\right) } (\Phi ({\tilde{C}}^{{{{\mathscr {R}}}}} \left( z\right) ,{\tilde{C}}\left( z\right) ))$ is the derivative of $\Phi ({\tilde{C}}^{{{{\mathscr {R}}}}} \left( z\right) ,{\tilde{C}}\left( z\right) )$, and ${{\mathscr {P}}}(v({\tilde{C}}^{{{{\mathscr {R}}}}} \left( z\right) ),v({\tilde{C}}\left( z\right) ))$ is a projection

72 $$\begin{aligned} {{{\mathscr {P}}}}(v({\tilde{C}}^{{{{\mathscr {R}}}}} \left( z\right) ),v({\tilde{C}}\left( z\right) ))=I-V(v({\tilde{C}}^{{{{\mathscr {R}}}}} \left( z\right) ),v({\tilde{C}}\left( z\right) ))V^{T} (v({\tilde{C}}^{{{{\mathscr {R}}}}} \left( z\right) ),v({\tilde{C}}\left( z\right) )), \end{aligned}$$

where *I* is the identity operator, while

73 $$\begin{aligned} V\left( v({\tilde{C}}^{{{{\mathscr {R}}}}} \left( z\right) ),v({\tilde{C}}\left( z\right) )\right) ={\textstyle \frac{v({\tilde{C}}\left( z\right) )-v({\tilde{C}}^{{{{\mathscr {R}}}}} \left( z\right) )}{\left\| v({\tilde{C}}\left( z\right) )-v({\tilde{C}}^{{{{\mathscr {R}}}}} \left( z\right) )\right\| }} . \end{aligned}$$

### Estimation error

Let assume that the physical reference measurement rounds is set to $$R_{{{{\mathscr {R}}}}}^{*} =R^{\left( \kappa \right) } $$ to evaluate $${\tilde{C}}^{{{{\mathscr {R}}}}} \left( z\right) $$, such that $$R^{*} $$ is the actually performed physical layer measurement rounds to evaluate $${\tilde{C}}\left( z\right) $$.

To measure the impacts of measurement rounds on the precision of the objective function estimation, we introduce the term $\mu _{\kappa } ({\tilde{C}}^{{{{\mathscr {R}}}}} \left( z\right) ,{\tilde{C}}\left( z\right) )$ that quantifies the mean squared error (MSE) at a particular scaling factor $\kappa$ as

74 $$\begin{aligned} \begin{aligned} \mu _{\kappa } \left( {\tilde{C}}^{{{{\mathscr {R}}}}} \left( z\right) ,{\tilde{C}}\left( z\right) \right) ={\textstyle \frac{1}{\kappa ^{2} R^{*} }} \sum _{i=0}^{\kappa ^{2} R^{*} -1}\left( C^{r,\left( i\right) } -C^{\left( i\right) } \right) ^{2} . \end{aligned} \end{aligned}$$

As the value of the $\kappa$ scaling factor increases, the information about the reference objective function ${\tilde{C}}^{{{{\mathscr {R}}}}} \left( z\right)$ increases, and the $\mu _{\kappa } ({\tilde{C}}^{{{{\mathscr {R}}}}} \left( z\right) ,{\tilde{C}}\left( z\right) )$ value decreases.

Then, let $$\mu _{1} ({\tilde{C}}^{{{{\mathscr {R}}}}} \left( z\right) ,{\tilde{C}}\left( z\right) )$$ be the MSE value obtainable at $$R^{*} $$ measurement rounds, i.e., $$\kappa =1$$, evaluated via as (74)

75 $$\begin{aligned} \mu _{1} \left( {\tilde{C}}^{{{{\mathscr {R}}}}} \left( z\right) ,{\tilde{C}}\left( z\right) \right) ={\textstyle \frac{1}{R^{*} }} \sum _{i=0}^{R^{*} -1}\left( C^{r,\left( i\right) } -C^{\left( i\right) } \right) ^{2} . \end{aligned}$$

For $$\kappa >1$$, let

76 $$\begin{aligned} \xi _{\kappa } =\sum _{i=R^{*} }^{\kappa ^{2} R^{*} -1}\left( C^{r,\left( i\right) } -C^{\left( i\right) } \right) ^{2} \end{aligned}$$

be a quantity that measures the squared difference of the objective function values. Assuming an optimal situation, the value of $\xi _{\kappa }$ is close to zero, $\xi _{\kappa } \approx 0$. For $\xi _{\kappa } =0$, it can be concluded that

77 $$\begin{aligned} \begin{aligned} \mu _{\kappa } \left( {\tilde{C}}^{{{{\mathscr {R}}}}} \left( z\right) ,{\tilde{C}}\left( z\right) \right)&={\textstyle \frac{1}{\kappa ^{2} }} \mu _{1} \left( {\tilde{C}}^{{{{\mathscr {R}}}}} \left( z\right) ,{\tilde{C}}\left( z\right) \right) \\ {}&={\textstyle \frac{1}{\kappa ^{2} }} \left( {\textstyle \frac{1}{R^{*} }} \sum _{i=0}^{R^{*} -1}\left( C^{r,\left( i\right) } -C^{\left( i\right) } \right) ^{2} \right) , \end{aligned} \end{aligned}$$

while for $$\xi _{\kappa } >0,$$

78 $$\begin{aligned} {\mu _{\kappa }} \left( {{\tilde{C}^{{\mathcal{R}}}} \left( z \right),\tilde{C}\left( z \right)} \right) = & \tfrac{1}{{{\kappa ^{2}} {R^{*}}}}\left( {\sum\limits_{{i = 0}}^{{R^{*} - 1}} {\left( {C^{{r,\left( i \right)}} - C^{{\left( i \right)}} } \right)^{2} + \xi _{\kappa } } } \right) \\  = & \tfrac{1}{{\kappa ^{2} }}\left( {\tfrac{1}{{R^{*} }}\left( {\sum\limits_{{i = 0}}^{{R^{*} - 1}} {\left( {C^{{r,\left( i \right)}} - C^{{\left( i \right)}} } \right)^{2} } } \right) + \tfrac{1}{{R^{*} }}\xi _{\kappa } } \right) \\  = & \tfrac{1}{{\kappa ^{2} }}\left( {\mu_{1} \left( {{\tilde{C}^{{\mathcal{R}}}} \left( z \right),\tilde{C}\left( z \right)} \right) + \tfrac{1}{{R^{*} }}\xi _{\kappa } } \right)\quad \\  = & \tfrac{1}{{\kappa ^{2} R^{*} }}\left( {\sum\limits_{{i = 0}}^{{R^{*} - 1}} {\left( {C^{{r,\left( i \right)}} - C^{{\left( i \right)}} } \right)^{2} + \sum\limits_{{i = {R^{*}} }}^{{\kappa ^{2} {R^{*}} - 1}} {\left( {C^{{r,\left( i \right)}} - C^{{\left( i \right)}} } \right)^{2} } } } \right) \\ \end{aligned} $$

that is, for $\xi _{\kappa } >0$, (78) coincidences with (74). Additional results are included in the Appendix.

### Cost reduction

The cost reduction is evaluated as follows. Let $$f_{0} $$ be the cost function of the evaluation of the reference objective function $${\tilde{C}}^{{{{\mathscr {R}}}}} \left( z\right) $$ via $$R_{{{{\mathscr {R}}}}}^{*} =R^{\left( \kappa \right) } $$ physical measurement rounds (i.e., no post-processing is applied), defined as a reference cost with a unit value

79 $$\begin{aligned} f_{0} =1. \end{aligned}$$

At a given $$f_{0} $$, the $$f\left( \kappa \right) $$ be the cost function associated to the evaluation of $${\tilde{C}}\left( z\right) $$ at a particular $$\kappa $$ and $$\xi _{\kappa } $$ is defined as

80 $$\begin{aligned} f\left( \kappa ,\xi _{\kappa } \right) =f_{0} \eta \left( \kappa ,\xi _{\kappa } \right) . \end{aligned}$$

where $\eta \left( \kappa ,\xi _{\kappa } \right)$ identifies the ratio of

81 $$\begin{aligned} \begin{aligned} \eta \left( \kappa ,\xi _{\kappa } \right)&={\textstyle \frac{\mu _{\kappa } \left( {\tilde{C}}^{{{{\mathscr {R}}}}} \left( z\right) ,{\tilde{C}}\left( z\right) \right) }{\mu _{1} \left( {\tilde{C}}^{{{{\mathscr {R}}}}} \left( z\right) ,{\tilde{C}}\left( z\right) \right) }} \\&={\textstyle \frac{{\textstyle \frac{1}{\kappa ^{2} }} \left( \mu _{1} \left( {\tilde{C}}^{{{{\mathscr {R}}}}} \left( z\right) ,{\tilde{C}}\left( z\right) \right) +{\textstyle \frac{1}{R^{*} }} \xi _{\kappa } \right) }{\mu _{1} \left( {\tilde{C}}^{{{\mathscr {R}}}} \left( z\right) ,{\tilde{C}}\left( z\right) \right) }} \\ {}&=\left( {\textstyle \frac{1}{\kappa ^{2} }} +{\textstyle \frac{1}{\left( \kappa ^{2} \mu _{1} \left( {\tilde{C}}^{{{{\mathscr {R}}}}} \left( z\right) ,{\tilde{C}}\left( z\right) \right) \right) }} \left( {\textstyle \frac{1}{R^{*} }} \xi _{\kappa } \right) \right) . \end{aligned} \end{aligned}$$

As follows, at $\xi _{\kappa } =0$, the proposed post-processing method reduces the cost of objective function estimation by a factor

82 $$\begin{aligned} \eta \left( \kappa ,0\right) ={\textstyle \frac{1}{\kappa ^{2} }} , \end{aligned}$$

and for any $\xi _{\kappa } >0$, the $\Delta f\left( \kappa ,{{\xi }_{\kappa }} \right)$ increment in the $f\left( \kappa ,0 \right)$ cost function is

83 $$\begin{aligned} \Delta f\left( \kappa ,{{\xi }_{\kappa }} \right) = \eta \left( \kappa ,\xi _{\kappa } \right) -\eta \left( \kappa ,0\right) ={\textstyle \frac{1}{\left( \kappa ^{2} \mu _{1} \left( {\tilde{C}}^{{{{\mathscr {R}}}}} \left( z\right) ,{\tilde{C}}\left( z\right) \right) \right) }} \left( {\textstyle \frac{1}{R^{*} }} \xi _{\kappa } \right) . \end{aligned}$$

**Figure 2:**
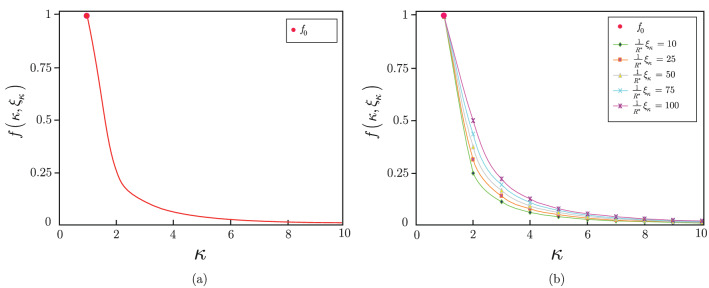
Cost reduction of objective function estimation. (a) The $f\left( \kappa ,\xi _{\kappa } \right)$ cost function at $\xi _{\kappa } =0$. The resulting cost is $f\left( \kappa ,0\right) ={\textstyle \frac{1}{\kappa ^{2} }}$. The initial objective function $f_{0} =1$ associated with the evaluation of the reference objective function ${\tilde{C}}^{{{{\mathscr {R}}}}} \left( z\right)$ from $R_{{{{\mathscr {R}}}}}^{*} =R^{\left( \kappa \right) }$ physical measurement rounds is depicted by a red dot. (b) The $f\left( \kappa ,\xi _{\kappa } \right)$ cost function at $\xi _{\kappa } >0$ scenarios at $\mu _{1} ({\tilde{C}}^{{{\mathscr {R}}}} \left( z\right) ,{\tilde{C}}\left( z\right) )=100$ and ${\textstyle \frac{1}{R^{*} }} \xi _{\kappa } =\left\{ 10,25,50,75,100\right\}$. The resulting cost is $f\left( \kappa ,\xi _{\kappa } \right) =f_{0} \eta \left( \kappa ,\xi _{\kappa } \right)$.

In Fig. 2. the $f\left( \kappa ,\xi _{\kappa } \right)$ cost function values are depicted for a given $\kappa$, $\kappa =\left\{ 1,\ldots ,10\right\}$, with $f_{0} =1$. In Fig. 2(a), the $\xi _{\kappa } =0$ scenario is depicted. In this case, the objective function estimation cost is reduced to $f\left( \kappa ,0\right) ={\textstyle \frac{1}{\kappa ^{2} }}$. In Fig. 2(b), the $\xi _{\kappa } >0$ scenario is illustrated for $\mu _{1} ({\tilde{C}}^{{{{\mathscr {R}}}}} \left( z\right) ,{\tilde{C}}\left( z\right) )=100$ and ${\textstyle \frac{1}{R^{*} }} \xi _{\kappa } =\left\{ 10,25,50,75,100\right\}$. The resulting cost is reduced to $f\left( \kappa ,\xi _{\kappa } \right) =f_{0} \eta \left( \kappa ,\xi _{\kappa } \right)$, where $\eta \left( \kappa ,\xi _{\kappa } \right)$ is as given in (81).

## Conclusion

Gate-model quantum computers provide an implementable architecture for experimental quantum computations. Here we studied the problem of objective function estimation in gate-model quantum computers. The proposed framework utilizes the measurement results and increases the precision of objective function estimation and maximization via computational steps. The method reduces the costs connected to the physical layer such as quantum state preparation, quantum computation rounds, and measurement rounds. We defined an objective function extension procedure, a segmentation algorithm that utilizes the gate parameters of the unitaries of the quantum computer, and a machine-learning unit for the system state prediction. The results are particularly convenient for the performance optimization of experimental gate-model quantum computers and near-term quantum devices of the quantum Internet.

### Ethics statement

This work did not involve any active collection of human data.

## Supplementary information

Supplementary Information

## Data Availability

This work does not have any experimental data.
