# Supplementary material for: Objective function estimation for solving optimization problems in gate-model quantum computers
Source: Sci Rep. 2020 Aug 26;10:14220. doi: 10.1038/s41598-020-71007-9 (PMC7450069; doi:10.1038/s41598-020-71007-9)
Supplement: Supplementary file 1 — Supplementary Information [file 41598_2020_71007_MOESM1_ESM.pdf]

Objective Function Estimation for Solving Optimization Problems in Gate-Model Quantum Computers

Laszlo Gyongyosi[1,2,3,*]

[1]School of Electronics and Computer Science, University of Southampton, Southampton, SO17 1BJ, UK
[2]Department of Networked Systems and Services, Budapest University of Technology and Economics, Budapest, H-1117 Hungary
[3]MTA-BME Information Systems Research Group, Hungarian Academy of Sciences, Budapest, H-1051 Hungary
[*]gyongyosi@hit.bme.hu

ABSTRACT

Quantum computers provide a valuable resource to solve computational problems. The maximization of the objective function of a computational problem is a crucial problem in gate-model quantum computers. The objective function estimation is a high-cost procedure that requires several rounds of quantum computations and measurements. Here, we define a method for objective function estimation of computational problems in gate-model quantum computers. The proposed solution reduces the costs of the objective function estimation and provides an optimized estimate of the state of the quantum computer for solving optimization problems.

A Appendix

A.1 Estimation Error

The actual objective function values and the related parameter values serve illustration purposes.

In Fig. A.1(a), a distribution of a set $\mathscr{S}\left\{\tilde{C}_i(z)\right\}_{i=1}^{6}$ of observed objective function values $\tilde{C}_i(z)$ are depicted at a particular reference objective function value $\tilde{C}^{\mathscr{R}}(z)$. In Fig. A.1(b), the values of $\Phi(\tilde{C}^{\mathscr{R}}(z), \tilde{C}(z))$ are depicted in function of the absolute value of the difference of $\tilde{C}^{\mathscr{R}}(z)$ and $\tilde{C}_i(z)$, $i=1,\ldots,6$.

Fig. A.1(c-d). illustrates the $\mu_\kappa\left(\tilde{C}^{\mathscr{R}}(z), \tilde{C}(z)\right)$ MSE values for $\kappa=\{1,\ldots,10\}$. In Fig. A.1(c) the ideal case, i.e., $\frac{1}{R^*}\xi_\kappa=0$ is depicted. In Fig. A.1(d), the non-ideal cases are depicted for $\frac{1}{R^*}\xi_\kappa=\{10,25,50,75,100\}$.

A.2 Notations

The notations of the manuscript are summarized in Table A.1.

Table A.1. Summary of notations.

Notation	*Description*
C	Objective function of a computational problem.
θ	Refers to the collection $\theta_1,\ldots,\theta_{N_{tot}}$, where θ_j is a continuous parameter.
$\lvert\theta\rangle$	Quantum state of the quantum computer dominated by computational basis states with a high value of an objective function C.
QG	Quantum gate structure of the quantum computer.
D_{QG}	Depth of the quantum circuit QG of the quantum computer.
M	Measurement.
N_{tot}	Total number of quantum gates of the QG quantum gate structure of the quantum computer.

U_j	A jth unitary of the QG quantum gate structure of the quantum computer, $j = 1, \ldots, N_{tot}$.
$\|\psi_0\rangle$	Initial state of the quantum computer.
$\|\psi_0\rangle$	Initial state of the quantum computer.
$f(\theta)$	Objective function of the quantum computer, $f(\theta) = \langle \theta \| C \mid \theta \rangle$,<br>where C is a classical objective function of a computational problem.
$N_G(U_j)$	Qubit number associated to gate U_j.
φ_j	Gate parameter of an $N_G(U_j)$-qubit unitary $U_j(\varphi_j)$, with relation<br>$U_j(\varphi_j) = U_j(\theta_j) = U(B_j, \varphi_j) = \exp(-i\varphi_j B_j)$,<br>where B_j is a set of Pauli operators.
B_j	Set of Pauli operators associated with a jth unitary U_j of the QG quantum gate structure, $j = 1, \ldots, N_{tot}$.
$\|z\rangle$	Computational basis.
$N(\varphi_j)$	Total number of occurrences of gate parameter value φ_j in the QG quantum circuit of the quantum computer.
$f^{(0)}(\theta)$	Estimate of function $f(\theta)$ at R^* physical measurement rounds.
$f^{(\kappa)}(\theta)$	Estimate of function $f(\theta)$ at $R^{(\kappa)}$ imaginary measurement rounds in the post-processing.
R	Number of measurement rounds.
R^*	Total number of physical measurements rounds.
$R^{(\kappa)}$	Number of imaginary measurement rounds, $R^{(\kappa)} = \kappa^2 R^*$.
κ	A scaling coefficient, $\kappa \geq 1$, defined as $\kappa = \sqrt{\frac{R^{(\kappa)}}{R^*}}$, where R^* is the total number of physical measurements, and $R^{(\kappa)} \geq R^*$ is the "imaginary" measurement rounds of the post-processing.
ξ_κ	A quantity that measures the squared difference of the objective function values $C^{r,(i)}$ and $C^{(i)}$, defined as $\xi_\kappa = \sum_{i=R^*}^{\kappa^2 R^* - 1} \left(C^{r,(i)} - C^{(i)} \right)^2$, where $C^{r,(i)}$ is the reference objective function in the ith physical measurement round, while $C^{(i)}$ is the objective function of the ith round.
$\tilde{C}(z)$	Averaged objective function.
$C^{(i)}(z)$	Objective function associated to the ith measurement round.
$\|M\|$	Total number of required measurements to get the estimate $f^{(0)}(\theta)$ at R rounds, $\|M\| = Rn$.
$\mathscr{F}$	Optimization framework.
θ^*	New value of θ.
$\|\theta^*\rangle$	New quantum state produced by the quantum computer.
$\mathscr{P}(\theta)$	Prediction for the selection of the new value of θ for the QG quantum gate structure.
n	Number of measurement gates in a round of M.
$\tilde{C}^0(z)$	Averaged objective function at R^* rounds.
$C^0(z)$	Cumulative objective function at R^* rounds.

$C^0(x,y)$	Component of $C^{(0)}(z)$ obtainable by the measurement of the yth qubit, $y=0,\ldots,n-1$, in the xth measurement round, $x=0,\ldots,R^*-1$.
$\varphi_{QG_R}(i,j)$	A φ gate parameter associated to the (i,j)-th gate, $i=0,\ldots,D_{QG}-1$, $j=0,\ldots,n-1$, of a reference quantum circuit QG_R.
$\varphi_{QG}^{\vec{\phi}^*}(i,j)$	A φ gate parameter associated to the (i,j)-th gate of the segmented QG circuit.
$C^{(i)}(z)$	Objective function of the ith round, $i=0,\ldots,R^*-1$, at R^* rounds.
$C^E(z)$	Extended objective function.
$d_{C^0(z)}$	Dimension of $C^0(z)$, $d_{C^0(z)}=(R^*\times n)$.
$d_{C^{(i)}(z)}$	Dimension of $C^{(i)}(z)$, $d_{C^{(i)}(z)}=(R^*\times n)$.
$d_{C^E(z)}$	Dimension of $C^E(z)$, $d_{C^E(z)}=\left(\kappa^2R^*\times n\right)$.
$\mathscr{A}_E$	Objective function extension algorithm.
$\mathscr{A}_D$	Quantum gate structure segmentation algorithm.
$\mathscr{A}_f^L$	Quantum gate parameter randomization algorithm, where L is the application level of $\mathscr{A}_f$.
$\mathscr{R}$	Rule generation algorithm.
$\mathscr{W}$	Discrete wavelet transform.
$\mathscr{W}^{-1}$	Inverse discrete wavelet transform function.
$f_\phi(\cdot)$	Wavelet basis function.
$W^{(j)}(z)$	Transformed objective function, $j=0,\ldots,w^{(l)}-1$.
$W^E(z)$	Extended transformed objective function.
S_1	Subset of $W^E(z)$, $S_1=\sum_{j=0}^{w^{(l)}-4}W^{(j)}(z)$ of dimension $d_{S_1\kappa}=\left(\frac{\kappa^2}{4}R^*\times n\right)$.
S_2,S_3,S_4	Subsets of $W^E(z)$ of dimension $d=\frac{1}{3}\left(\left(\kappa^2R^*\times n\right)-d_{S_1\kappa}\right)$.
$w^{(l)}$	A parameter of $\mathscr{W}$. Number of transformed objective function values at a given level l, $l\geq 1$, expressed as $w^{(l)}=4+3(l-1)$.
P_E	Sub-procedure 1 of Algorithm 1 ($\mathscr{A}_E$).
$\mathscr{W}^*(z)$	A maximized value of the results of $\mathscr{W}$ operators,<br>$\mathscr{W}^*(z)=\max_{\forall i}\mathscr{W}^i(z)$,<br>where $i=0,\ldots,l-1$, and $d_{\mathscr{W}^*(z)}=d_{\mathscr{W}^i(z)}=(R^*\times n)$.
$\lambda_E(\cdot)$	Objective function extension factor.
n_t	Number of classes selected for the segmentation of the φ gate parameters of the QG structure of the quantum computer in algorithm $\mathscr{A}_D$.
H_k	Entropy function associated to the kth class, $k=1,\ldots,n_t$ in algorithm $\mathscr{A}_D$.
$f(\vec{\phi})$	Objective function of the segmentation algorithm $\mathscr{A}_D$ of the QG quantum gate structure.
ϕ_l	Gate segmentation parameter for to the classification of the φ gate parameters into lth and $(l+1)$-th classes, $0\leq\phi_l\leq\chi$, where χ is an upper bound on the gate parameters of the quantum computer.
$\vec{\phi}$	An $d_{\vec{\phi}}=(n_t-1)$-dimensional vector $\vec{\phi}=[\phi_1,\ldots,\phi_{n_t-1}]$.
$\vec{\phi}^*$	Optimal vector that maximizes $f(\vec{\phi})$, $\vec{\phi}^*=\arg\max_{\vec{\phi}}f(\vec{\phi})$.

$N(\varphi_i)$	Number of occurrences of φ_i in the QG structure of the quantum computer.
$\Pr(N(\varphi_i))$	Probability distribution, $\Pr(N(\varphi_i)) = \frac{N(\varphi_i)}{N_{tot}}$, where N_{tot} is the total number of quantum gates in the QG quantum circuit of the quantum computer, $\sum_{i=1}^{N_{tot}} \Pr(N(\varphi_i)) = 1$.
ω_i	Sum of probability distributions.
$\mathscr{C}_{QG}$	Classes of the segmentation procedure, $\mathscr{C}_{QG} : \{\mathscr{C}_1, \ldots, \mathscr{C}_{n_t}\}$.
μ_{QG}	Class mean values of $\mathscr{C}_{QG}$, $\mu_{QG} : \{\mu_1, \ldots, \mu_{n_t}\}$.
$\mathscr{C}_{QG} \in \varphi_j$	Classification of gate parameter φ_j into $\mathscr{C}_{QG}$.
D	Iteration level of $\mathscr{A}_D$.
X,Y	Numbers from the range of $[0,1]$, $X,Y \in [0,1]$.
q,w,p	Uniform random numbers from the range of $[0,1]$, $q,w,p \in U[0,1]$.
ξ	Control parameter in $\mathscr{A}_D$.
S	Search space.
$\underline{S}(j)$,$\overline{S}(j)$	Lower and upper bounds on the search space S.
N_i	Iteration number for algorithm $\mathscr{A}_D$.
$\varepsilon_{\vec{\varphi}^*}$	Error associated to the gate parameter segmentation algorithm $\mathscr{A}_D$.
$\mathscr{I}(\mathscr{A}_f^{j+1})$	Input set in algorithm $\mathscr{I}(\mathscr{A}_f^{j+1}) = \mathscr{C}_t(\mathscr{A}_f^j)$.
r	Ratio parameter in $\mathscr{A}_f^L$, $r \in [0,1]$.
$\mathscr{S}_l^v$	Random learning set of size $r\lvert\mathscr{S}_{\mathscr{A}_f^L}\rvert$.
$\mathscr{S}_t^v$	Random test set $\mathscr{S}_t^v$ of size $(1-r)\lvert\mathscr{S}_{\mathscr{A}_f^L}\rvert$.
$\mathscr{R}_{\mathscr{S}_l}$	Rule set, $\mathscr{R}_{\mathscr{S}_l} = \{R_1, \ldots, R_L\}$ for procedure $\mathscr{R}$.
R_i	An ith rule.
$A(R_i)$	Rule antecendant for R_i.
$C(R_i)$	Consequence of rule R_i.
$\mathscr{L}(R_i)$	Leverage of R_i.
$\alpha(\cdot)$	Coverage function.
$S(R_i)$	Single rule $S(R_i) = \{A(R_i), C(R_i)\}$.
R_v^*	Optimal rule with highest leverage coefficient.
$\mathscr{R}_{\mathscr{S}_l}^*$	Set of optimal rules $\mathscr{R}_{\mathscr{S}_l}^* = \{R_1^*, \ldots, R_L^*\}$ for procedure $\mathscr{R}$.

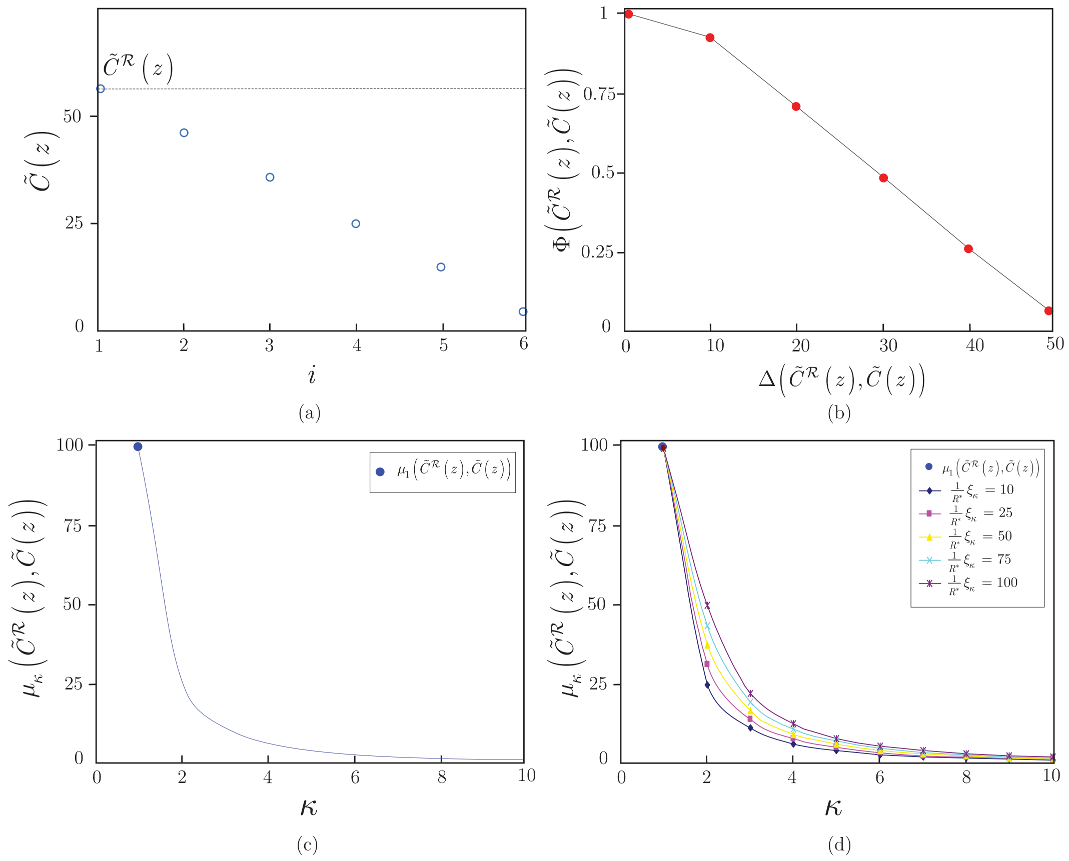

Figure A.1. (a) Distribution of the $\tilde{C}_i(z)$ objective function values in $\mathscr{S}\{\tilde{C}_i(z)\}_{i=1}^{6}$. The objective function values are set to $\tilde{C}_i(z) = \tilde{C}^{\mathscr{R}}(z) - 10(i-1)$, $i = 1, \ldots, 6$ (depicted by blue line dots), where the $\tilde{C}^{\mathscr{R}}(z)$ reference objective function is set to $\tilde{C}^{\mathscr{R}}(z) = 53.6$ (dashed line). The $\tilde{C}^{\mathscr{R}}(z)$ reference objective function is parameterized with standard deviation $\sigma_{\tilde{C}^{\mathscr{R}}(z)} = 91.1314$, while set $\mathscr{S}\{\tilde{C}_i(z)\}_{i=1}^{6}$ is parameterized with standard deviation set $\mathscr{S}\{\sigma_{\tilde{C}_i(z)}\}_{i=1}^{6}$, as $\sigma_{\tilde{C}_1(z)} = \sigma_{\tilde{C}^{\mathscr{R}}(z)}$, $\sigma_{\tilde{C}_2(z)} = 101.9064$, $\sigma_{\tilde{C}_3(z)} = 121.1905$, $\sigma_{\tilde{C}_4(z)} = 145.6420$, $\sigma_{\tilde{C}_5(z)} = 173.0845$ and $\sigma_{\tilde{C}_6(z)} = 202.3046$, while set $\mathscr{S}\{\sigma_{\tilde{C}^{\mathscr{R}}(z)\tilde{C}_i(z)}\}_{i=1}^{6}$ is as $\sigma_{\tilde{C}^{\mathscr{R}}(z)\tilde{C}_1(z)} = 8304.9333$, $\sigma_{\tilde{C}^{\mathscr{R}}(z)\tilde{C}_2(z)} = 8789.3778$, $\sigma_{\tilde{C}^{\mathscr{R}}(z)\tilde{C}_3(z)} = 9273.8222$, $\sigma_{\tilde{C}^{\mathscr{R}}(z)\tilde{C}_4(z)} = 9758.2666$, $\sigma_{\tilde{C}^{\mathscr{R}}(z)\tilde{C}_5(z)} = 10242.7111$ and $\sigma_{\tilde{C}^{\mathscr{R}}(z)\tilde{C}_6(z)} = 10727.1555$. (b) The distribution of $\Phi(\tilde{C}^{\mathscr{R}}(z), \tilde{C}(z))$ values for $\mathscr{S}\{\tilde{C}_i(z)\}_{i=1}^{6}$ (depicted by red dots) at $\tilde{C}^{\mathscr{R}}(z)$, in function of $\Delta(\tilde{C}^{\mathscr{R}}(z), \tilde{C}(z)) = |\tilde{C}^{\mathscr{R}}(z) - \tilde{C}_i(z)|$, $i = 1, \ldots, 6$. (c) An ideal case. The $\mu_\kappa(\tilde{C}^{\mathscr{R}}(z), \tilde{C}(z))$ values at $\frac{1}{R^*}\xi_\kappa = 0$ for $\kappa = \{1, \ldots, 10\}$, with $\mu_1(\tilde{C}^{\mathscr{R}}(z), \tilde{C}(z)) = 100$ (depicted by the blue dot). (d): Non-ideal cases. The values of $\mu_\kappa(\tilde{C}^{\mathscr{R}}(z), \tilde{C}(z))$ at $\frac{1}{R^*}\xi_\kappa > 0$ for $\kappa = \{1, \ldots, 10\}$, $\frac{1}{R^*}\xi_\kappa = \{10, 25, 50, 75, 100\}$, $\mu_1(\tilde{C}^{\mathscr{R}}(z), \tilde{C}(z)) = 100$.
